# Mathematical analysis of emicizumab: affinity-driven complex formation and lipid-surface reactions

**DOI:** 10.1016/j.jtha.2025.07.002

**Published:** 2025-07-16

**Authors:** Jamie Madrigal, Dougald M. Monroe, Suzanne S. Sindi, Karin Leiderman

**Affiliations:** 1 Mathematics Department, University of North Carolina at Chapel Hill, Chapel Hill, North Carolina, USA; 2 Department of Medicine, University of North Carolina at Chapel Hill, Chapel Hill, North Carolina, USA; 3 UNC Blood Research Center, University of North Carolina at Chapel Hill, Chapel Hill, North Carolina, USA; 4 Department of Applied Mathematics, University of California Merced, Merced, California, USA; 5 Computational Medicine Program, University of North Carolina at Chapel Hill, Chapel Hill, North Carolina, USA; 6 Department of Biochemistry and Biophysics, University of North Carolina at Chapel Hill, Chapel Hill, North Carolina, USA

**Keywords:** bispecific antibody, emicizumab, hemophilia A, mathematical modeling

## Abstract

**Background:**

Emicizumab is a bispecific antibody that binds activated factor (F)IXa with 1-arm and FX with the other. Binding bridges FIXa and FX, replacing the function of FVIII in hemophilia A. Unlike FVIII, emicizumab does not bind directly to lipid surfaces.

**Objectives:**

This study aimed to investigate emicizumab’s lipid-surface dependent mechanisms through mathematical modeling and biochemical assays.

**Methods:**

We expanded our mathematical model of tissue factor (TF):VIIa activation of FX to incorporate emicizumab and FIXa interactions. We calibrated our model using experimental data.

**Results:**

High concentrations of emicizumab inhibit FX activation by TF:VIIa. Our mathematical model explains these observations only when FX bound to emicizumab is partially restricted from binding to lipid surfaces and to TF:VIIa. Lipid enhances FX activation of FIXa in the presence of emicizumab. In our 2-arm interaction model, we estimated kinetic rates for emicizumab-dependent activation of FX on the lipid surface. The model successfully predicted FIXa activation of FX with and without emicizumab across many experimental conditions. Ternary complexes (FIXa, FX, and emicizumab) in solution decreased when lipid increased while ternary complexes on lipid increased. Sensitivity analysis, which varied lipid, dissociation constants, and catalytic rates, highlighted the impact of binding-arm affinity on reaction velocities.

**Conclusion:**

High concentrations of emicizumab decrease TF:VIIa activation of FX by reducing FX binding to both the lipid surface and TF:VIIa. Emicizumab enhances FIXa activation of FX on the lipid surface by preferentially binding to lipid-bound FX and subsequently to lipid-bound FIXa with an enhanced association rate due to colocalization on the lipid surface.

## 1 | INTRODUCTION

Hemophilia A is an X-linked bleeding disorder characterized by a deficiency in coagulation factor (F)VIII [1]. This deficiency results in prolonged and unwanted bleeding, which can range from mild to severe. The standard of care in the United States is prophylactic replacement therapy, where recombinant or plasma-derived FVIII is administered to replace the deficient FVIII [2]. Compliance with replacement therapy is often limited due to the need for multiple weekly intravenous (i.v.) injections, driven by the short half-life of FVIII (8–12 hours) [2]. Treatment with extended half-life FVIII reduces dosing frequency, but still requires i.v. injections [3,4].

**FIGURE 1:**
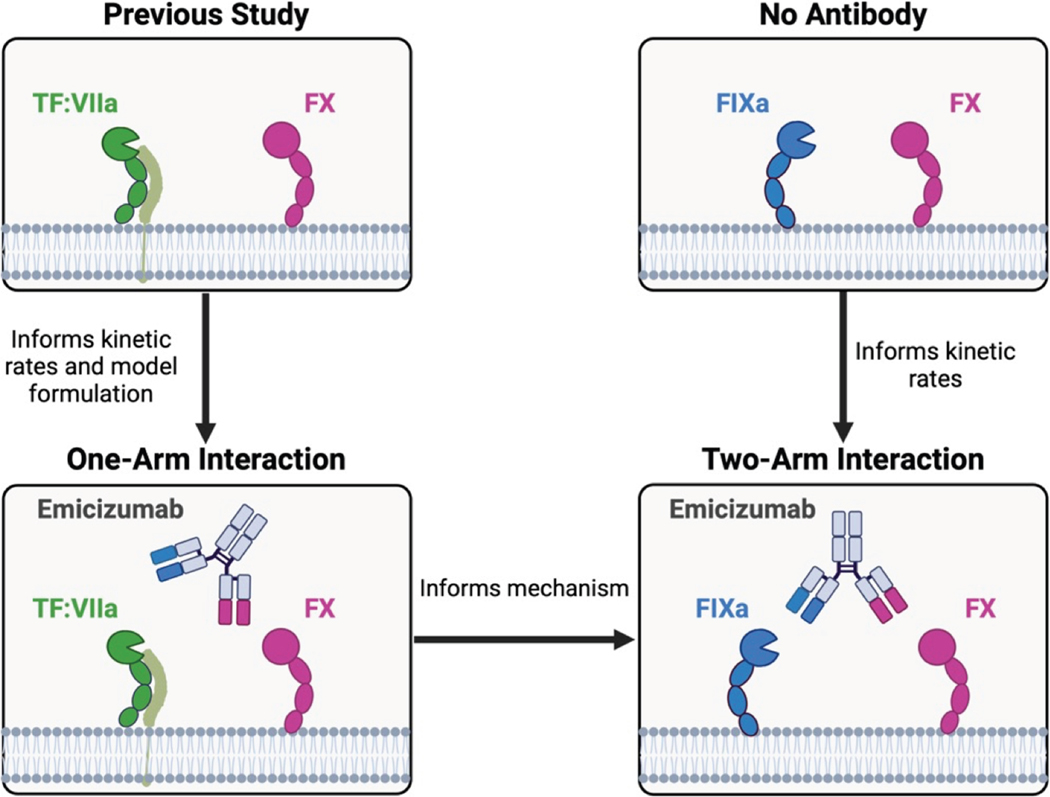
Schematic of project workflow. We expanded our previous mathematical model of tissue factor (TF):VIIa activation of factor (F) X to include emicizumab (1-arm model). We estimated kinetic rates and schemes of the 1-arm model to experimental data. Parameter estimates from the 1-arm model were carried forward to the 2-arm model. Additional experiments and modeling of FIXa, FX, and lipid informed the kinetic rates for the 2-arm model.

Another approach to treating hemophilia A involves the use of hemostatic agents that replace the function of deficient FVIII. One such agent is emicizumab, a bispecific monoclonal antibody designed to bridge factor (F)X with activated FIXa for efficient activation [5,6]. Emicizumab binds FIX or FIXa with 1 antigen-binding fragment and FX and FXa with the other (Figure 1; 2-arm interaction). Treatment with emicizumab requires subcutaneous injections every 1 to 4 weeks [7] and is not recognized by FVIII inhibitors [4]. Emicizumab’s high bioavailability and long half-life (4–5 weeks) [2] further enhance its effectiveness in facilitating activation of FX [5].

Although emicizumab is a widely used US Food and Drug Administration-approved therapeutic, questions remain regarding its precise mechanisms of action. Emicizumab replaces only part of FVIIIa activity. A key mechanistic difference is that emicizumab does not bind directly to the cell surface, whereas FVIII does. The binding of FVIII(a) to the lipid surface localizes and stabilizes FXa generation on the surface. In the presence of lipid, the affinity of FVIIIa and FIXa increases by 2000-fold [8]. Surface-bound FVIIIa binds to surface-bound FIXa, inducing a conformational change in the FIXa protease domain [9], which enhances the catalytic activity of the enzyme-cofactor complex by 1500-fold [10]. The surface localizes the enzyme and cofactor to the same site and positions the enzyme-cofactor complex for efficient FX activation. The binding of FVIIIa to the surface enables it to function at a much lower concentration than emicizumab [11]. While emicizumab does not bind directly to lipid, FIXa and FX must bind to a lipid surface to form a ternary complex with emicizumab [5,12]. It is hypothesized that surface binding is required for proper alignment of the enzyme and substrate [6]. Moreover, emicizumab binds both precursor and activated proteins, such as FIX and FIXa, and does not require activation, unlike FVIII. It is therefore always “on.” Consequently, FXa generation is limited not by the amount of FVIIIa but by the amount of FIXa [6].

Different techniques used to measure the binding affinities of emicizumab with FIX/FIXa and FX/FXa yield conflicting results [5,12], contributing to the uncertainty surrounding emicizumab. Kitazawa et al. [5] report similar binding affinities of emicizumab to FIX/FIXa ($K_{\text{D}}=1.5$ μM) and FX/FXa ($K_{\text{D}}=1.8$ μM), while Mak et al. [12] observed a higher affinity between emicizumab and FX ($K_{\text{D}}=56$ nM) than emicizumab and FIX ($K_{\text{D}}=5.5$ μM). These differing rates suggest distinct mechanisms underlying emicizumab’s efficiency. Kitazawa et al. [5] propose that most FIXa and FX exist as monomers, implying that an abundant concentration of emicizumab is required for sufficient ternary complex formation. In contrast, Mak et al. [12] suggest that the fast dissociation rate of emicizumab from FIX relative to that from FX enhances its efficiency by freeing the enzyme to activate additional zymogen. Regardless of binding affinity, the catalytic efficiency ($K^{\text{cat}}$) of emicizumab is approximately 10-fold lower than that of FVIIIa [6]. This discrepancy raises the question of how emicizumab effectively replaces FVIIIa despite its (1) weak affinity for FIX/FIXa and FX/FXa, (2) reduced catalytic efficiency, and (3) inability to bind the lipid surface.

In this study, we aimed to determine emicizumab’s mechanism of action using an interdisciplinary approach combining biochemical assays and mathematical modeling. As illustrated in Figure 1, we structured the mathematical and experimental study to progressively explore the full interaction between emicizumab, FIXa, and FX through a series of experiments with nested components. While previous studies have used mathematical approaches to model bispecific antibodies [13–15], to our knowledge, we have developed the first mathematical models of emicizumab.

Our biochemical assays confirmed that emicizumab requires lipid for FX activation by FIXa and revealed that emicizumab has an inhibitory effect on FX activation by tissue factor (TF):VIIa. These findings motivated us to conduct a mathematical study of the one-arm interaction between emicizumab and FX in the absence of FIXa to uncover the inhibitory mechanism. We developed a mathematical model of TF:VIIa activation of FX in the presence of lipid and emicizumab, building on our previously validated model of TF:VIIa and FX on a lipid surface [16]. Using experimental data to guide mathematical modeling assumptions, we identified that emicizumab-bound proteins have reduced binding to the lipid surface.

As a control, we analyzed FIXa, FX, and lipid alone before introducing emicizumab and developed the corresponding mathematical model to estimate kinetic rates for the enzymatic reaction in the absence emicizumab. Next, we conducted experiments with FIXa, FX, emicizumab, and lipid, varying the concentrations of emicizumab. We then developed the corresponding mathematical model and estimated the remaining unknown kinetic parameters.

Our mathematical models accurately captured the experimental data and suggest that emicizumab hinders proteins bound to it from directly interacting with lipid surfaces and with TF:VIIa on the lipid surface. Using our models, we determined the optimal efficacy of emicizumab in our experimental context, by varying its dissociation constants with FIX/FIXa and FX/FXa, as well as the catalytic rate of FIXa activation. Our results inform the design of future bispecific antibodies and encourage further investigation of off-target effects of emicizumab.

## 2 | METHODS

### 2.1 | Experimental procedures

Factors IXa, X, and VIIa were purchased from Prolytix (Essex Junction). Factor Xa was removed from FX by treatment with a mixture of dansyl-Glu-Gly-Arg chloromethylketone and phenylmethylsulfonyl fluoride (MilliporeSigma). Factor X was repurified on a HiTrap Q HP column (Cytiva). After binding FX, the column was washed with 250 nM NaCl in 20 mM HEPES (pH 7.4). Factor X was eluted with 10 mM CaCl_2_ in 20 mM HEPES (pH 7.4) and 250 mM NaCl. Calcium was removed by dialysis. Emicizumab was the generous donation of anonymous patients. Recombinant apo-TF was obtained from Millipore-Sigma. Phospholipids were obtained from Avanti Polar Lipids and prepared as large unilamellar vesicles as previously described [17]. Briefly, lipids were dried from chloroform under nitrogen, taken up in cyclohexane, lyophilized overnight, taken up in 20 mM HEPES (pH 7.4), 150 mM NaCl, put through 3 freeze–thaw cycles, sonicated 5 times for 1 minute each time in an ice water bath, and extruded through a 0.22-mμ filter. The vesicles consisted of 20 mol% phosphatidylserine, 40 mol% phosphatidylethanolamine, and 40 mol% phosphatidylcholine. Apo-TF was incorporated by incubation into vesicles as previously described by Krishnaswamy [18]. Factor Xa substrate (methoxycarbonyl-D-cyclohexylalanyl-glycylarginine para-nitroanilide) was purchased from PentaPharm.

Buffer was 20 mM HEPES (pH 7.4), 150 mM NaCl, 0.2% polyethylene glycol 8000, and 5 mM CaCl_2_. For assays studying FVIIa activation of FX, 25 μL of FVIIa was added to 25 μL of TF/lipid and incubated for 5 minutes. Factor X was incubated with emicizumab and FXa substrate. Fifty microliters of FX/emicizumab/substrate was added to FVIIa/TF/lipid to give the indicated concentrations. Substrate cleavage was monitored at 405 nm in a Molecular Devices ThermoMax microplate reader.

For assays studying FIXa activation of FX with the concentration of emicizumab varied, emicizumab was mixed with FIXa. Separately, FX was mixed with lipid and substrate for FXa. Further, 50 μL of FIXa/emicizumab was added to 50 μL of FX/lipid/substrate. Substrate cleavage was monitored at 405 nm. For assays in the absence of lipid, the same protocol was followed except that FIXa concentration was increased to give a final concentration of 10 nM. For assays in which emicizumab was held constant but FX was varied, emicizumab was mixed with FX. Separately, FIXa was mixed with lipid and substrate for FXa. Further, 50 μL of FX/emicizumab was added to 50 μL of FIXa/lipid/substrate. Substrate cleavage was monitored at 405 nm.

### 2.2 | Mathematical model of 1-arm interaction between emicizumab and FX

**FIGURE 2:**
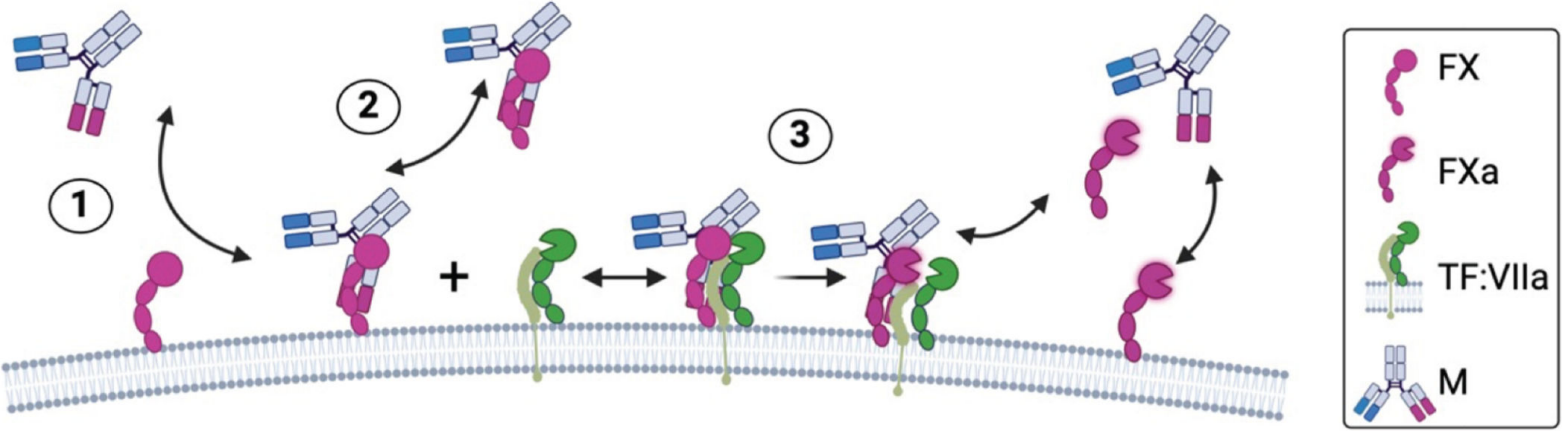
Schematic of emicizumab (M) involvement in tissue factor (TF):VIIa activation of factor (F)X. (1) M in solution binding with lipid-bound FX. (2) M bound to FX in solution binding to lipid. (3) TF:VIIa activation of FX bound to M.

The first mathematical model we developed represents the 1-arm interactions between emicizumab and FX. The model consists of our previously published model of TF:VIIa activation of FX in the presence of lipid surfaces [16], with emicizumab included. In this scenario, we assumed emicizumab (M) could work through 3 different possible, but not mutually exclusive, mechanisms: (1) by binding directly to lipid-surface bound FX/FXa; (2) by binding to solution-phase FX/FXa and consequently the M:X/Xa complex binds to the lipid surface; (3) by enabling activation of FX by TF:VIIa while bound to FX (Figure 2). The complete list of biochemical reactions and kinetic rate constants is given in Supplementary Table S1.

### 2.3 | Example equation

Biochemical reactions were translated into ordinary differential equations using the law of mass action. For example, the equation describing the rate of change of solution-phase FX is as follows:

$$\frac{dX}{dt}=-k_{X}^{on}XL^{+}+k^{off}X^{b,+}-k_{X}^{on}XL^{-}+k_{X}^{off}X^{b,-}-k_{MX}^{+}XM+k_{MX}^{-}M:X,$$

where X is the concentration of solution-phase FX, $L^{+}$ and $L^{-}$ are the concentrations of lipid with and without TF:VIIa, respectively, and $X^{b,+}$ and $X^{b,-}$ are the concentrations of FX bound to $L^{+}$ and $L^{-}$, respectively, and M is emicizumab. The kinetic rates for FX binding to/from lipid are denoted with the superscripts on/off, while the association/dissociation of FX with emicizumab have superscripts +/−. Complexes comprised of 2 model species (eg, M and FX) are denoted with a “:” between the model species (M:X).

### 2.4 | Mechanism testing with the mathematical model of the 1-arm interaction

We used the mathematical model of the TF:VIIa activation of FX in the presence of emicizumab and lipid to determine the underlying inhibitory mechanism. We call $Xa^{sim}$ the total concentration of FXa calculated with the model simulations and $Xa^{exp}$ the experimentally measured value. The goodness of fit of the model to the data is quantified by a relative sum of squared error as follows:

(1) $$\mathrm{Error}\left(Xa^{sim}|Xa^{exp}\right)=\sum_{i=1}^{n_{M}}\sum_{j=1}^{n_{t}}{\left(\frac{Xa_{i,j}^{exp}-Xa_{i,j}^{sim}}{Xa_{i,j}^{sim}}\right)}^{2},$$

where $n_{M}$ and $n_{t}$ are the number of emicizumab concentrations and number of time points used in the experiments, respectively.

### 2.5 | Mathematical model of the 2-arm interaction

**FIGURE 3:**
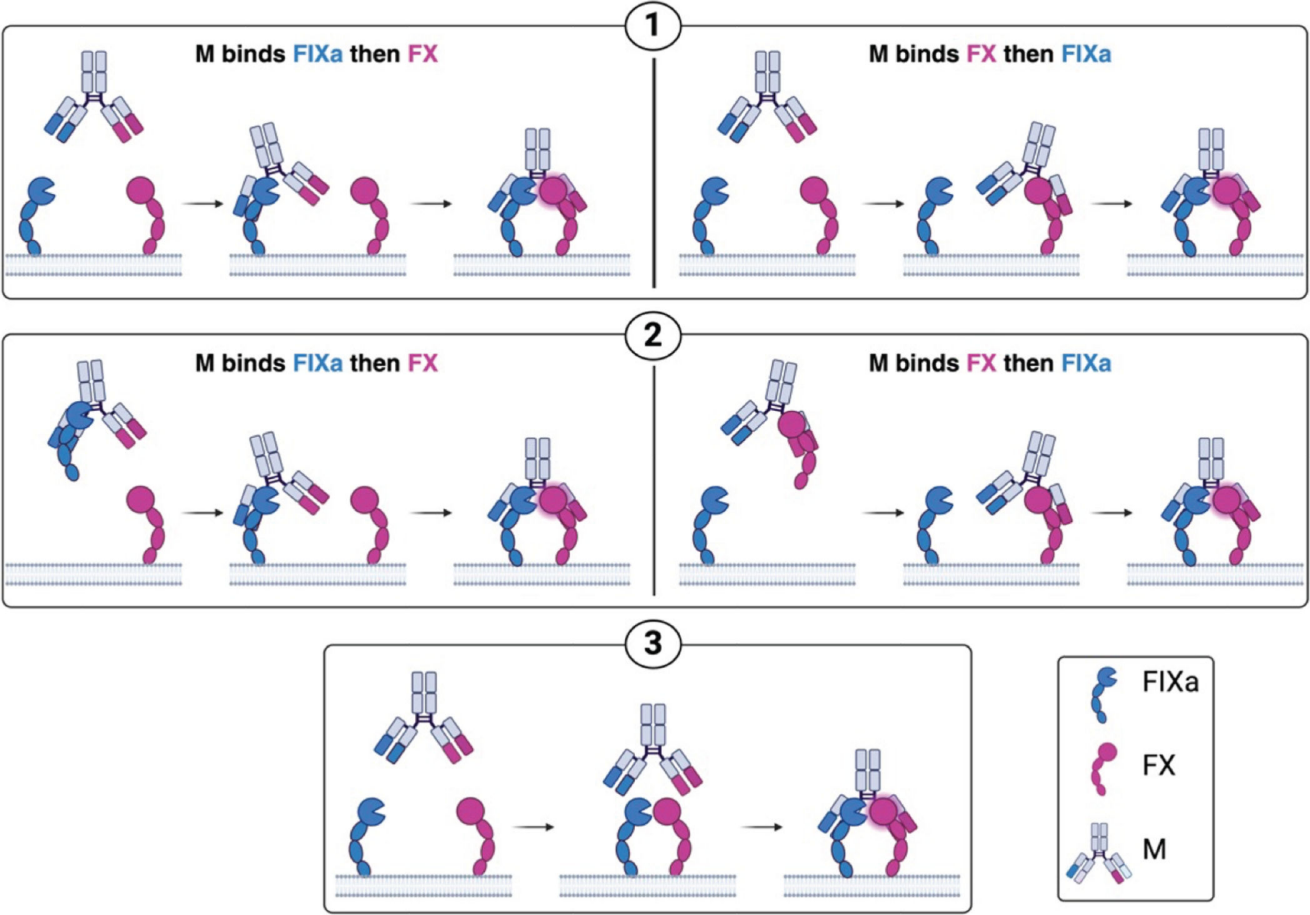
Schematic of ternary complex formation of emicizumab (M), factor (F)IXa, and FX. (1) M binds to 1 lipid-bound protein and then the complex binds the other lipid-bound protein. (2) M binds protein in solution and then the complex binds lipid, which then binds the other protein. (3) M binds directly to the lipid-bound FIXa:FX complex.

The mathematical model of the 2-arm interaction builds on the model of the 1-arm interaction. It differs by removing TF:VIIa and adding FIXa. We used experimental data to guide the model’s development and ensure that the necessary interactions introduced by FIXa were included. We considered 3 possible reactions between FIXa, FX/FXa, lipid, and emicizumab (M) (Figure 3). We assumed that FX activation occurred exclusively on the lipid surface, as suggested by previous studies [6] and supported by experiments in the current study. We also included FIXa activation of FX in the absence of emicizumab [19].

The 2-arm interactions in our experiments involved formation of a ternary complex with FIXa, emicizumab, and FX/FXa. We assumed that formation of this complex could follow any or all of the 3 pathways: (1) emicizumab binds lipid-bound FIXa (or FX) and subsequently lipid-bound FX (or FIXa), (2) emicizumab binds solution-phase FIXa (or FX) and FIXa (or FX) in that complex subsequently binds lipid and then the complex binds lipid-bound FX (or FIXa), and (3) emicizumab binds the lipid-bound FIXa–FX complex (Figure 3). Upon formation of the ternary complex with FX, FIXa activates FX to FXa. The ternary complex dissociates when emicizumab unbinds either FIXa or FX. The list of all biochemical reactions scheme is given in Supplementary Table S2. The reactions are transformed to a system of ordinary differential equations assuming mass action kinetics, as described for the 1-arm interaction model.

### 2.6 | Estimation of unknown kinetic parameters

We used Bayesian inference to estimate unknown kinetic parameters, treating them as random variables with associated probability densities. First, we found an initial starting guess for the unknown parameters, then we used a Markov chain Monte Carlo (MCMC) algorithm to estimate the posterior probability density. To select a starting guess for our MCMC, we used Latin hypercube sampling to generate hundreds of initial parameter values, assuming uniform distributions within prescribed lower and upper bounds for each. We calculated the error between the model and experimental data with each of these parameter combinations and selected the one with smallest error for further study. To further optimize this choice before running the MCMC algorithm, we used the previously chosen combination as the starting point for MATLAB’s function fmincon (a gradient-based constrained optimization method), which then determined the parameters that minimized the error function. This optimized set of parameters served as the initial condition for the MCMC algorithm. We used the MCMC toolbox package developed for MATLAB, that employs the delayed rejection adaptive metropolis algorithm [20,21].

The parameters estimated in the 1-arm mathematical model were chosen to minimize the error as described in Equation 1. Factor Xa generation through FIXa activation was measured with a chromogenic substrate as described earlier. To compare our simulations with experiments, we tracked the cleavage of the chromogenic substrate, denoted C:S, by FXa. S is the substrate that binds FXa and C is the chromophore concentration measured in experiments. A full set of chromogenic substrate reactions is in Supplementary Table S2. We denote $C_{i,j,k}^{exp}$ as the experimentally measured concentration for the specified emicizumab concentration, FX concentration, and time point, and $C_{i,j,k}^{sim}(\theta )$ as the corresponding simulated chromophore concentration generated with the parameter set, θ. We chose the optimal parameter set as the one that minimized the error between the simulated and experimental chromophore concentration:

(2) $$\mathrm{Error}\left(\theta |C^{sim},C^{exp}\right)=\sum_{i=1}^{n_{M}}\sum_{j=1}^{n_{FX}}\sum_{k=1}^{n_{t}}{\left(\frac{C_{i,j,k}^{exp}-C_{i,j,k}^{sim}(\theta )}{C_{i,j,k}^{sim}(\theta )}\right)}^{2},$$

where $n_{M}$, $n_{FX}$, and $n_{t}$ are the number of emicizumab concentrations, FX concentrations, and time points, respectively.

## 3 | RESULTS

### 3.1 | Emicizumab enhances FX activation by FIXa but reduces FX activation by TF:VIIa

**FIGURE 4:**
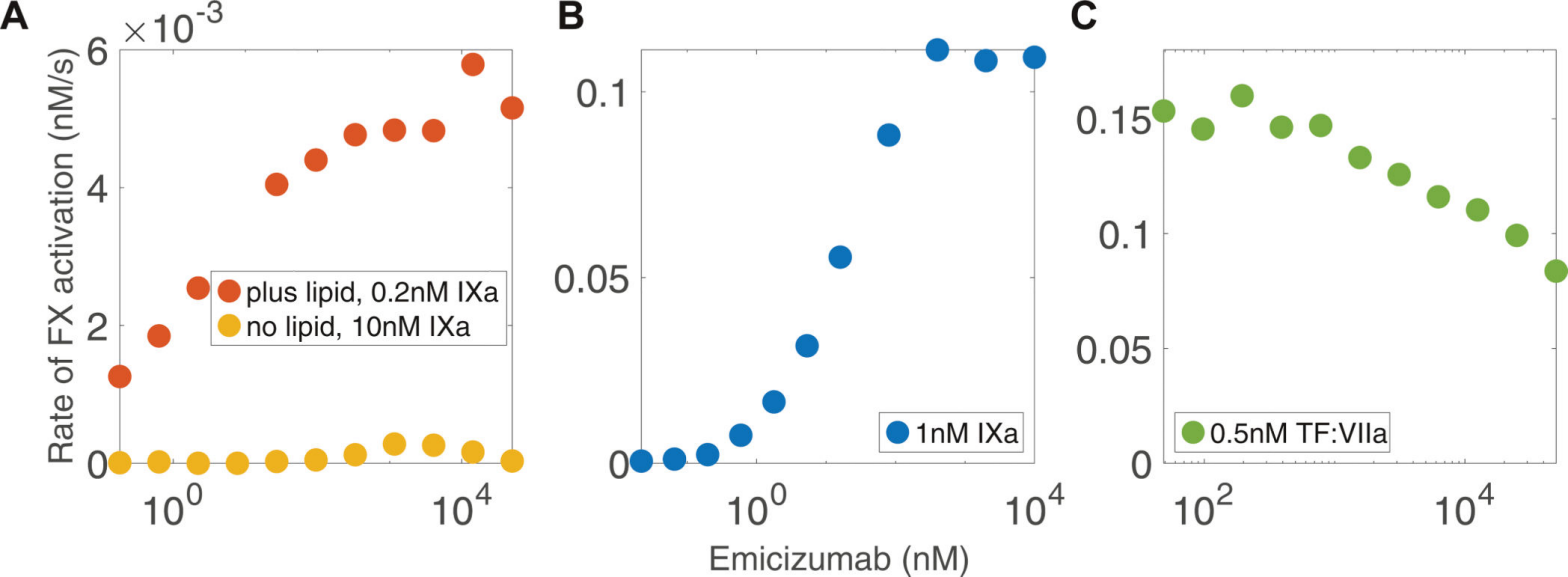
Experimental rate of factor (F)X activation as a function of emicizumab concentration. (A) FX (140 nM) activation by FIXa with lipid (80 μM) at 0.2 nM FIXa (blue) and without lipid at 10 nM of FIXa (orange). (B) FX (140 nM) activation by FIXa with lipid (80 μM) at 1 nM of FIXa. (C) Tissue factor (TF):VIIa activation of FX (140 nM) with lipid (80 μM) at 0.5 nM of TF:VIIa.

Emicizumab functions as a bispecific antibody that facilitates FX activation by FIXa. Its mechanism of action in the presence of lipid surfaces and its potential interference with FX activation by TF:VIIa has not yet been characterized. We therefore measured FX activation by FIXa and TF:VIIa while systematically varying lipid and emicizumab concentrations. Concentrations were chosen to be consistent with previous experiments [16]. We found that lipid surfaces were essential for FX activation by FIXa in the presence of emicizumab, even at relatively high FIXa concentrations (10 nM) (Figure 4A). While emicizumab enhanced the rate of FX activation by FIXa (Figure 4B), it unexpectedly reduced FX activation by TF:VIIa (Figure 4C); however, this reduction was observed only at high, nontherapeutic concentrations of emicizumab.

### 3.2 | Reduced TF:VIIa activity due to emicizumab-bound FX/FXa

The reduced TF:VIIa activity in the presence of emicizumab is due to its interactions with the FX/FXa binding arm of the antibody, as FIX/FIXa was not present. We used the 1-arm interaction model to explore this mechanism. We hypothesized that emicizumab reduces FX activation by TF:VIIa by either inhibiting FX/FXa from binding to lipid surface when bound to emicizumab or by inhibiting the binding of lipid-bound FX to TF:VIIa. These mechanisms are shown schematically in reactions (2) and (3), respectively, in Figure 2.

Our hypotheses were supported by additional experiments that measured FX activation by TF:VIIa for different FX concentrations with 0 and 50 μ M emicizumab (Supplementary Figure S2). The experimental results showed that emicizumab has little effect on the maximum velocity of the reaction but increases the apparent $K_{M}$. This increase in the apparent $K_{M}$ suggests that emicizumab does not alter the catalytic rate of the reaction but instead reduces the amount of FX available for activation. We hypothesized 2 possible mechanisms for this effect. First, emicizumab may interfere with FX binding to TF:VIIa. A recent study showed that FX interacts with TF:VIIa through its Gla and protease domain, while the FX light chain (epidermal growth factor [EGF]1, EGF2) does not directly contact TF:VIIa [22]. Since emicizumab binds the EGF2 domain of FX, it is unlikely to cause direct steric hindrance. However, it may still affect the rate of complex formation between FX and TF:VIIa, thereby slowing activation. Second, emicizumab may decrease the amount of available FX on the surface. To investigate this, we measured the concentration of FXa in solution after preincubation with lipid, both in the presence and in the absence of emicizumab (Supplementary Figure S1). We observed an increase in the fraction of FXa remaining in solution when emicizumab was present, indicating reduced binding of FXa bound to the lipid surface. This supports the idea that emicizumab decreases the lipid surface-bound pool of FX. To quantify these effects, we used the 1-arm mathematical model to evaluate how emicizumab influences binding to both TF:VIIa and the lipid surface.

**FIGURE 5:**
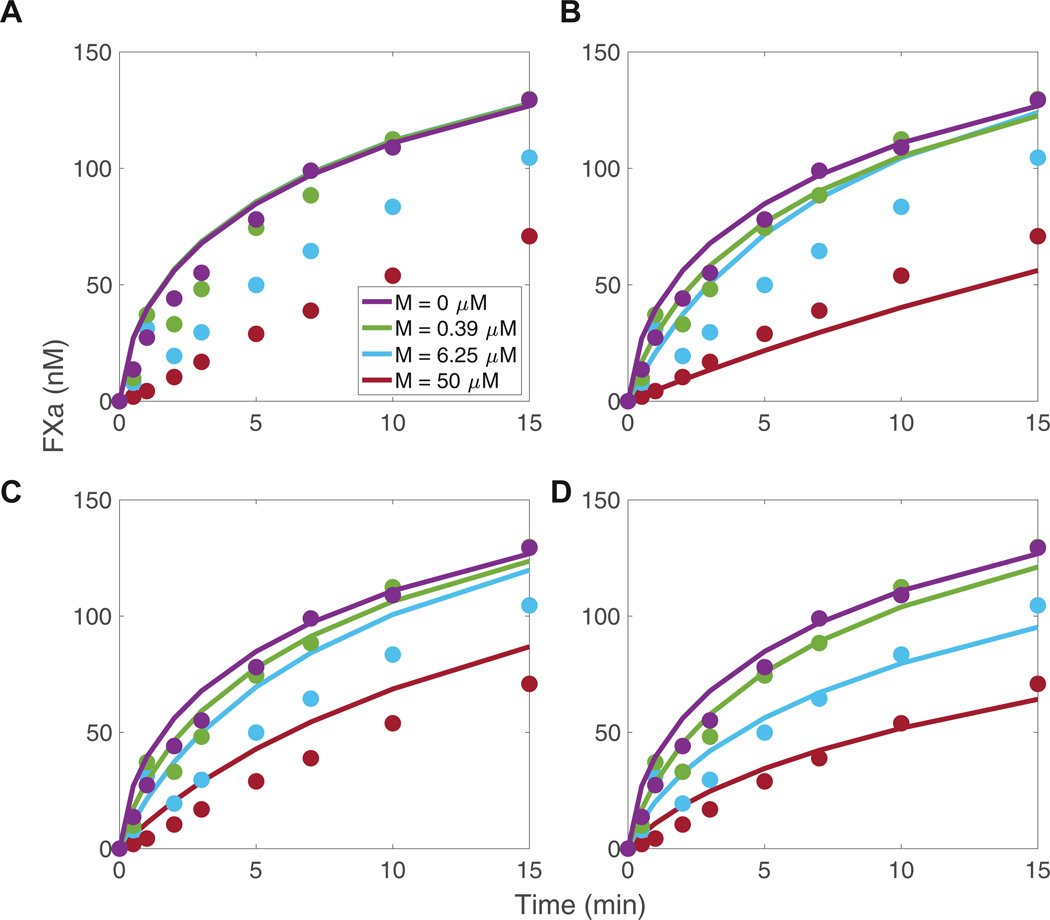
Factor (F)X activation by tissue factor (TF):VIIa in the presence of lipid and emicizumab (M). Each plot displays the same experimental measurements of FXa concentrations (dots) for varying concentrations of M (indicated by different colors), overlaid with simulations of FXa generated with the 1-arm mathematical model (solid lines). The simulations are based on 3 assumptions: (A) the kinetic rates of FX activation by TF:VIIa with and without emicizumab are equal (B) that emicizumab blocks lipid binding of FX/FXa, (C) that emicizumab blocks TF:VIIa binding of FX/FXa, and (D) that emicizumab inhibits both lipid and TF:VIIa binding of FX/FXa.

Figure 5 shows the time courses of FXa generated by TF:VIIa at 4 different concentrations of emicizumab. Emicizumab concentrations far above the therapeutic concentration [23] (400 nM) were studied to pinpoint the mechanism of the interaction. This is relevant for modeling of bispecific antibodies and reduces uncertainty in the larger, 2-arm model. The results for all concentrations are available in Supplementary Figure S5. Panels A–D display the FXa concentration simulated by the mathematical model under different assumptions. The dots represent experimentally measured FXa concentrations, while the solid lines represent the FXa concentration simulated by the model. The error between the experimentally measured and simulated concentrations was quantified by Equation (1). In the experiments, the rate of FXa generation decreased as the emicizumab increased from 0 to 50 μ M.

The mathematical model that assumed emicizumab does not alter the kinetic rates of FX activation by TF:VIIa (Supplementary Table S1) failed to reproduce the inhibitory behavior observed in the experimental data (Figure 5A), resulting in identical FXa generation at all concentrations of emicizumab. In contrast, the mathematical model that assumed emicizumab-bound FX/Xa could not bind lipid provided a slightly better fit to the data (Figure 5B), resulting in lower error between the simulated and experimentally measured FXa concentrations than the model under assumptions A and C. However, this model did not accurately capture FXa concentration at early timepoints. The model assuming emicizumab blocked the binding site of FX/Xa to TF:VIIa (Figure 5C) also failed to match experimental FXa generation, particularly at high emicizumab concentrations, where it overestimated the degree of inhibition.

Since neither assumption alone adequately explained the experimental results and because it is biologically unlikely that emicizumab completely blocks either the lipid binding of FX/Xa or the binding site of FX/Xa to TF:VIIa, we introduced 2 scaling parameters into the model. These parameters, α and β, were used to adjust the binding rate of FX/Xa to lipid and the association rate with TF:VIIa, respectively. We estimated the effect of both inhibitory mechanisms (Figure 5D) using parameter estimation methods described earlier. The model predicted that emicizumab reduced, but did not completely block, both the lipid binding and TF:VIIa binding of FX/Xa by about 50-fold (Supplementary Table S3).

To address discrepancies between 2 reported $K_{\text{D}}$ values for FX binding to emicizumab (1.56 μM from Kitazawa et al. [1] and 55 nM from Mak et al. [12]), we performed the parameter inference for α and β for each $K_{\text{D}}$ value and conducted a goodness-of-fit comparison (Supplementary Figures S3 and S4). The results strongly favored the Mak value, providing robust quantitative support for its use in our modeling. Additionally, using the Kitazawa value resulted in estimated parameters that suggested less biologically plausible behavior, including even more limited lipid binding and stronger TF:VIIa association (Supplementary Material). Based on this analysis, we proceeded with the Mak value in all subsequent simulations.

### 3.3 | The 2-arm interaction model: calibration by parameter estimation

Our experimental design allowed us to estimate a small number of parameters using a two-step approach. First, we used data collected in the absence of emicizumab to determine the kinetic rates for FX activation by FIXa on a lipid surface. Then, we used data from experiments with emicizumab present to estimate its kinetic rates. This approach enabled us to estimate parameters that Mak et al. [12] did not report. Since their study measured binding affinities for FIX rather than FIXa, their values may underestimate binding to emicizumab–FX complexes. Moreover, because Mak et al. [12] did not report rates for emicizumab–FXa and Kitazawa et al. [5] suggested emicizumab binds FX and FXa differently, uncertainty remains regarding the true binding affinity between emicizumab and FXa.

**FIGURE 6:**
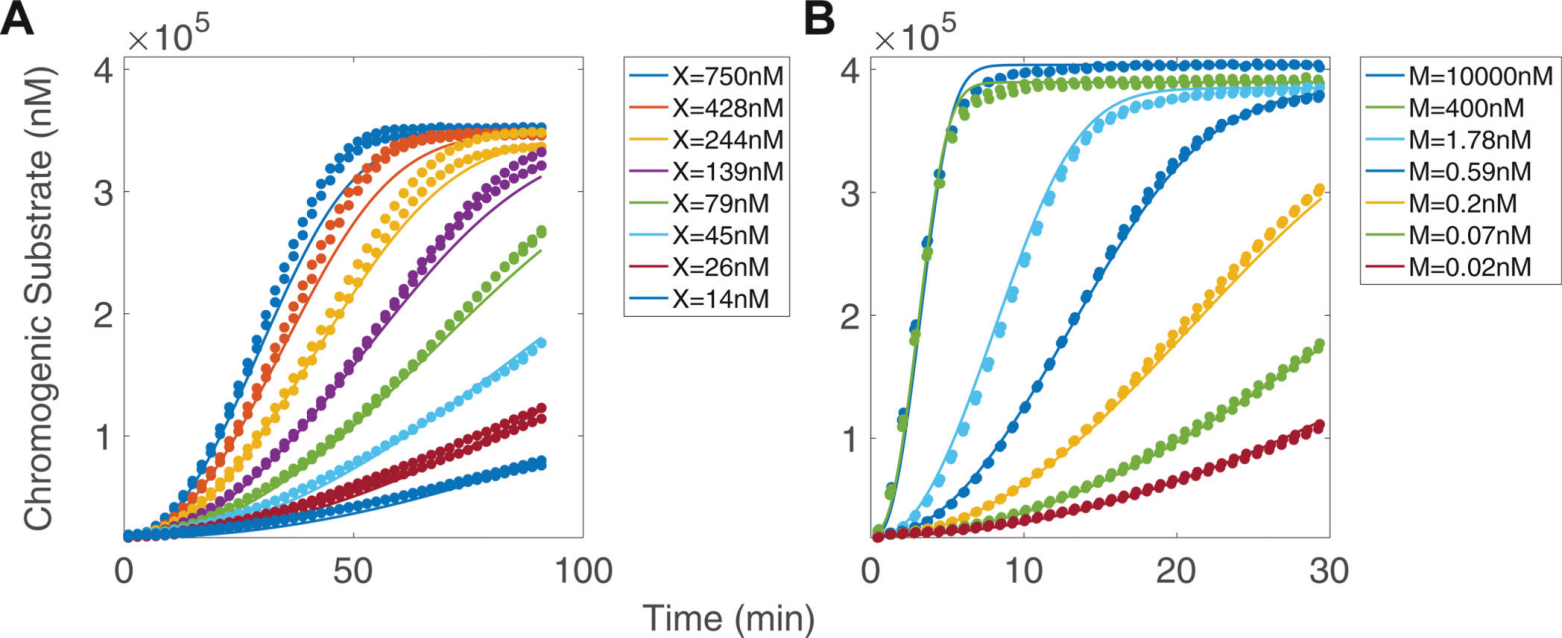
Factor (F)X activation by FIXa in the presence of lipid: no emicizumab, varied FX and fixed FX, and varied emicizumab. Each plot displays different experimental measurements of cleavage of FXa chromogenic substrate (dots, duplicate experiments), overlaid with simulations of chromogenic substrate cleavage generated with the 2-arm mathematical model (solid lines). (A) FX activation by FIXa (1 nM) with lipid (80 μM) without emicizumab under variations in FX concentration over 90 minutes. (B) FX (140 nM) activation by FIXa (1 nM) with lipid (80 μM) with emicizumab under variations in emicizumab concentration over 30 minutes.

In the absence of emicizumab, we estimated the dissociation constant $\left(K_{D}\right)$ and catalytic rate $\left(k^{cat}\right)$ for FX activation by FIXa to be 774 nM and 0.005/s, respectively. These estimations suggest a high dissociation rate between FIXa and FX and a slow catalytic rate compared with the reaction in the presence of FVIIIa [19]. The model and estimated parameters captured experimental data across many FX concentrations (Figure 6A). Note that the x-axes in Figure 6A and B are different; due to the slow activation of FX by FIXa, it was necessary to consider a longer time course for this reaction.

We estimated the remaining unknown parameters using experimental data from varying emicizumab with fixed FIXa, FX, and lipid concentrations. The model-simulated FXa concentrations were insensitive to changes in the association rate for IXa:M binding to FX, and thus, we assumed the rate reported for IX:M binding FX from Mak et al. [12]. In our experiments and simulations, the concentrations of emicizumab and FX were far more abundant than FIXa. Consequently, the concentration of IXa:M complexes was very low compared with that of M:X. Additionally, emicizumab binds FX tighter ($K_{D}=56$ nM) than FIXa ($K_{D}=5.5$ μM). Therefore, the ternary complex was primarily formed through lipid-bound FIXa binding the complex of emicizumab and lipid-bound FX.

The 1-arm interaction model and parameter estimation revealed that emicizumab reduced the binding of emicizumab-bound FX to the lipid surface and TF:VIIa on the lipid surface, through a ~50-fold reduction in association rate (Supplementary Table S3). Emicizumab may also lead to reduced lipid binding while bound to FIX/FIXa. Previous studies found that emicizumab does not affect FIX activation by TF:VIIa [24]; however, the concentration of emicizumab used in that study was close to the therapeutic dose, which is not high enough to see a reduction in TF:VIIa activity as our study suggests. Because we tested a wide range of emicizumab concentrations, we assumed that emicizumab-bound FIX/FIXa and FX/FXa exhibit similar reduced lipid binding, and thus we applied the α valued estimated from the 1-arm model to the association rate for both IXa/IXa:M and M:X/Xa binding to lipid.

The next step was to estimate the association rate for lipid-bound M:X binding to lipid-bound IXa. We estimated a higher association rate for FIXa (7.02 × 10^−1^/nM/s) than previously reported for FIX (1.27 × 10^−4^/nM/s) [12]. Additionally, the estimated dissociation rate for emicizumab and FXa increased compared to the rate for emicizumab and FX. Our Bayesian inference approach revealed a broad distribution of potential scaling factors for the dissociation rate (~10–1000) (Supplementary Figure S6A), indicating that the $K_{\text{D}}$ must be at least 10 times greater than that of X binding to emicizumab. Beyond this value, however, the model exhibits limited sensitivity to further increases. This implies that emicizumab quickly unbinds from newly activated FXa, allowing for further activation of FX. We also estimated the catalytic rate of FX activation by FIXa and emicizumab to be 0.485/s, which implies that emicizumab increases activation about 100-fold compared with FIXa alone but is not as efficient as FVIIIa [19]. Figure 6B shows the model simulations using the mean of the parameter distribution estimates (Supplementary Table S4), which provide an excellent fit to the data across many concentrations of emicizumab.

### 3.4 | The 2-arm interaction model: validation with a separate data set

We validated the 2-arm interaction model and the estimated parameters using a separate data set from the one used for calibration. In these validation experiments, we measured the cleavage of FXa substrate in the presence of lipid, with a fixed emicizumab concentration of 400 nM, and varied concentrations of FX. We performed forward simulations of the model using the parameters estimated in the previous section, and the results are shown in Supplementary Figure S7. The results demonstrated that the mathematical model, with the estimated kinetic rates, accurately captured FXa generation across all FX concentrations.

### 3.5 | Lipid-dependent ternary complex formation enhances emicizumab efficacy

**FIGURE 7:**
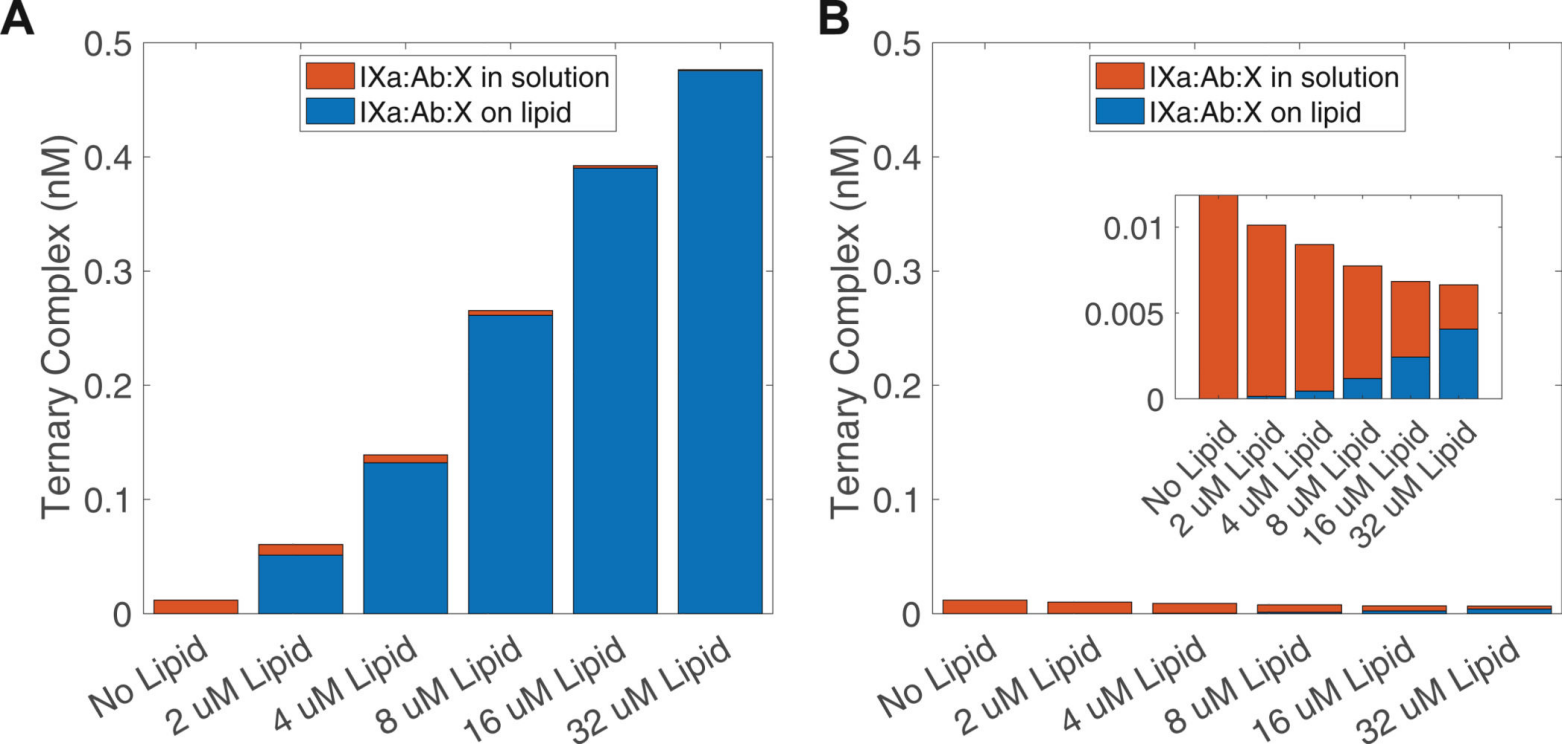
Proportions of solution-phase and lipid-bound ternary complexes as lipid concentration changes. (A) Simulated concentrations of solution-phase and lipid-bound ternary complexes (FIXa:M:FX) across increasing lipid concentrations (0–32 μM). (B) Simulations in which we decrease the association rate of lipid-bound FIXa to M:FX to be equal to that in solution. All concentrations were measured at 2 minutes. Simulations included 140 nM of FX, 1 nM of FIXa, and 400 nM of emicizumab.

Emicizumab exhibits significantly enhanced efficacy in the presence of lipid (Figure 4A). To investigate the mechanistic basis of this effect, we expanded the 2-arm interaction mathematical model to include solution-phase FX activation by the IXa and emicizumab complex (Supplementary Table S2). These reactions were not included when estimating parameters of the 2-arm interaction model as they are not significant at the lipid concentration (80 μM) studied. Ternary complex formation in solution, via either IXa binding to M:X or X binding to IXa:M, was modeled using the association and dissociation rates as those used for the binding of IXa and X to M individually. The catalytic rate for FX activation in solution was set to same value we estimated for the lipid-bound complex. We simulated FX activation across a range of lipid concentrations (0–32 μM), quantifying both solution-phase and lipid-bound ternary complexes and then compared their relative abundances (Figure 7A). In the absence of lipid, only ~0.01 nM IXa:M:X formed in solution, representing ~1% of total IXa after 2 minutes of simulation. As lipid concentration increased, total ternary complex formation rose sharply, reaching ~0.5 nM at 32 μM lipid—approximately 50% of total IXa. Notably, most of this complex was lipid bound, while the solution-phase complex diminished to near-zero levels. To assess the role of membrane localization, we repeated the simulation with a decreased association rate for lipid-bound IXa to M:X; we reduced the association rate from the value we estimated to the value we used for the solution-phase association. Under these conditions (Figure 7B), total ternary complex formation remained below 0.012 nM across all lipid concentrations. Although the proportion of lipid-bound complexes still increased with lipid concentration, the overall complex formation was substantially reduced. These findings indicate that although emicizumab partially inhibits lipid binding of proteins when bound to them, the enhanced association rate resulting from membrane colocalization is the key driver of emicizumab’s increased efficacy in lipid-rich environments.

### 3.6 | Influence of antibody binding-arm affinities and catalytic rates on reaction velocities

It is challenging to extract biochemical mechanisms from experimentally measured reaction velocities in complex biochemical systems that include surfaces, enzymes, cofactors, and substrates. These complexities are evident in reactions involving bispecific antibodies like emicizumab. For simplicity, we will hereafter refer to a general bispecific antibody as Ab. While a few binding affinities for Abs have been measured by others or estimated by us (as discussed earlier), only mathematical simulations can precisely estimate the velocity at which FX is cleaved while within the ternary complex attached to a lipid surface.

**FIGURE 8:**
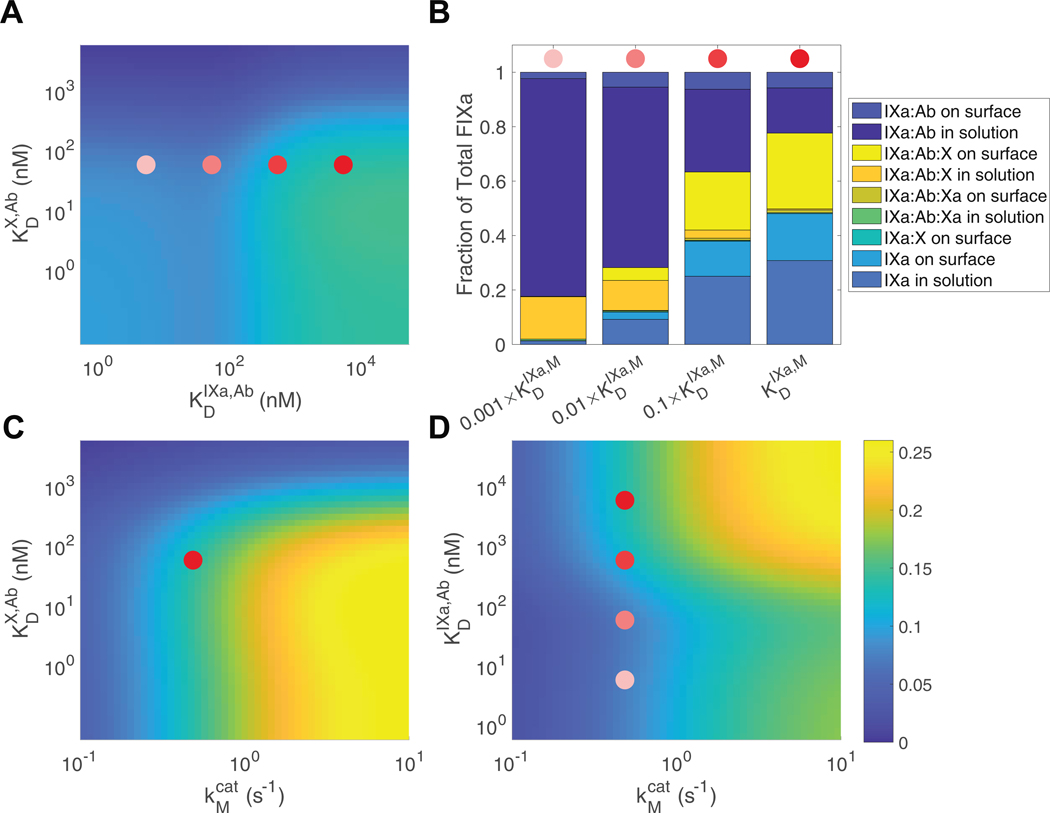
Model (2-arm)-simulated reaction velocity (rate of factor [F]X activation) with variation in model parameters. (A) Heatmap of the simulated reaction velocity as a function of $K_{D}^{IXa,Ab}$ and $K_{D}^{M,Ab}$. (B) Distribution of concentrations of all model species that contain FIXa at 2 minutes of simulated reaction time. Heatmaps of the simulated reaction velocity at 2 minutes of simulated reaction time as a function of $k^{cat}$ and $K_{D}^{IXa,Ab}$ (C) and $K_{D}^{X,Ab}$ (D). The red dots indicate dissociation constants for emicizumab, $K_{D}^{IXa,M}$ and $K_{D}^{X,M}$, containing our estimated kinetic rates. As the dots transition from red to white, the dissociation constant, $K_{D}^{IXa,M}$, decreases by a factor of 10, indicating increasingly tighter binding between FIXa and emicizumab. In all simulations, we assumed 8 μM of lipid, 1 nM of FIXa, 140 nM of FX, and 400 nM of Ab.

To understand how the reaction velocity of the FIXa within the ternary complex depends on binding affinities between FIXa and FX with an Ab, we used our 2-arm interaction model and systematically varied the dissociation rates for the binding arms of FIXa and FX. We computed the reaction velocity from the simulations as the initial slope (over 2 minutes) of the simulated FXa concentrations and reported these slopes as reaction velocities in the heatmaps shown in Figure 8. In these heatmaps, we varied the dissociation constants $K_{D}^{IXa,Ab}$, $K_{D}^{X,Ab}$ and the catalytic rate, $k^{cat}$, where 140 nM FX, 1 nM FIXa, 8 μM lipid, and 400 nM Ab are mixed together. Yellow indicates a high reaction velocity, while blue indicates a slow one. The red dot represents the dissocation constants we estimated for emicizumab with our experimental data, $K_{D}^{IXa,M}$ and $K_{D}^{X,M}$.

In Figure 8A, we varied $K_{D}^{X,Ab}$ and $K_{D}^{IXa,Ab}$ and found that the optimal reaction velocities occur when $K_{D}^{IXa,Ab}>K_{D}^{X,bsAb}$. We showed dots that transition in color from red to white, indicating that as the dissociation constant for FIXa and the Ab decreases by a factor of 10, relative to that for emicizumab $\left(K_{D}^{IXa,M}\right)$, the binding between FIXa and the Ab becomes increasingly tighter. As the binding gets tighter, between the red and white dot, the reaction velocity decreased about 2-fold. To determine the mechanism behind this decrease in velocity, we tracked the concentrations of every model species that included FIXa: FIXa in solution and bound to the surface, FIXa bound to FX on the surface, FIXa bound to the Ab in solution and on the surface, and FIXa bound in a ternary complex on the surface. After 2 minutes of reaction time, we recorded the distributions of these species as a fraction of the total FIXa (1 nM). The stacked bar chart in Figure 8B shows these distributions corresponding to dissociation constants marked with the same dots as in Figure 8A. We see that the distributions of FIXa vary greatly as the dissociation rate changes. When the binding is very tight between FIXa and the Ab, most of the FIXa is in IXa:Ab and IXa:Ab:X in solution. Since there is more FX and the dissociation rate for FX is fixed and tight for the study in Figure 8B, each of these scenarios should have sufficient FX bound to Ab on the surface and in solution. Note that the dissociation constant for FIXa and lipid is 190 nM. As the dissociation constant for FIXa to the Ab increases, the proportion of FIXa bound to the ternary complex (IXa:Ab:X) in solution decreases and the proportion of FIXa in the lipid-bound ternary complex increases, up to about a third of the total FIXa when the dissociation constant equals that of emicizumab. This is similar behavior to that when we increase the lipid concentration, leaving the dissociation constants fixed.

We also investigated how changing the catalytic rate of the ternary complex would alter the reaction rate for a variety of FX-binding and FIXa-binding arm dissociation constants. In all cases, increasing the $k^{cat}$ led to a monotonic increase in the reaction rate (Figure 8C, D). Specifically, we varied $k^{cat}$ from 0.01/s to 10/s, observing that higher catalytic rates consistently resulted in higher reaction velocities. This suggests that the catalytic efficiency of the ternary complex is a critical factor in determining the overall reaction rate. Although reaction velocities increase with lipid under varying dissociation rates (Supplementary Figure S8), our simulations show that increasing the catalytic rate is more effective than altering antibody arm affinities, an effect that becomes even more pronounced in the presence of lipid.

## 4 | DISCUSSION

Emicizumab is a widely used treatment for hemophilia A, but the mechanism of its efficiency is not well understood. Emicizumab replaces only part of the full FVIIIa activity. Under normal/healthy clotting conditions, the formation of the ternary complex (FVIIIa: FIXa:FX) is a multistep process and is limited by the amount of FVIIIa. The first step involves colocalization of the FVIIIa–FIXa complex on the lipid surface. Factor VIIIa binds lipid more efficiently than FIXa [25], which favors formation of the complex on the lipid surface compared to in solution. The lipid surface limits proteins to a 2-dimensional space and positions the FVIIIa–FIXa complex into an active conformation. The complex of FVIIIa–FIXa then binds lipid-bound FX. Since emicizumab cannot bind the lipid surface, it lacks the ability to directly colocalize FIXa and FX on the surface. Emicizumab does not distinguish between precursor and activated proteins and does not require activation like FVIIIa does. It is therefore always on. Consequently, FXa generation is no longer limited by the amount of FVIIIa, but the amount of enzyme, FIXa [6]. While this is beneficial to forming emicizumab-protein complexes, the low level of self-regulation, ie, emicizumab always being on, in combination with the long half-life could have downstream effects.

Our findings on emicizumab were possible only though our innovative and interdisciplinary approach. The study includes FX activation by 2 enzymes, TF:VIIa and FIXa. The 2-arm interaction of emicizumab with FIX/FIXa and FX/FXa involved many possible chemical reactions. This made it difficult to identify the primary mechanism of FX activation. The study of the 1-arm interaction allowed for simplification of the system and utilization of previously published work [16]. One limitation of these studies is that our experiments and simulations were conducted at a single lipid and FIXa concentration, meaning all reported kinetic rates are apparent rates. The lipid concentration was specifically chosen to minimize surface crowding and the template effect, as detailed in our previous work [16]. Since the reaction is limited by the amount of FIXa, the results may vary at different concentrations.

Although emicizumab is designed to enhance FX activation, it slowed activation by TF:VIIa at high concentrations (>3000 nM) (Figure 5). The concentrations tested are far above the therapeutic dose (~400 nM) but gave insight into the mechanisms of interaction. The 1-arm interaction model explained the experimental observations only if emicizumab-bound proteins were inhibited from binding to the surface, ie, FX/FXa-lipid binding, and to TF:VIIa.

This hypothesis was further supported by experiments (Supplementary Figure S1). Factor X binds tighter to emicizumab ($K_{\text{D}}=55$ nM [5]) than lipid ($K_{\text{D}}=190$ nM [12]) which implies there will be less lipid-bound FX when emicizumab is present. However, our modeling shows that the amount of lipid-bound FX is sufficient for emicizumab to bind it directly and then bind to the lipid-bound FIXa. Mathematical modeling was essential to pinpoint the mechanism of inhibition. Previous studies that utilized experimental data alone, and not mathematical models, could not explain with certainty the source of the observed inhibition [26].

Emicizumab’s binding to FX/FXa and FIX/FIXa partially restricts these complexes from binding lipid, making its efficiency even more puzzling. Simulations of the model revealed that the primary modality to ternary complex formation is through lipid-bound M:X binding lipid-bound FIXa, with an association rate that is greatly enhanced on the lipid surface compared with the one in solution. Given our hypothesis that emicizumab-bound proteins have reduced binding to lipid, and considering the lipid binding affinities of FIX/FIXa and FX/FXa, and the concentrations of proteins, it follows that emicizumab in solution is likely binding to proteins that are already bound to the lipid surface, allowing emicizumab to rapidly bind to lipid-bound FIXa. This could be beneficial in vivo since the concentration of FX is much higher than FIXa so that the large concentrations of FX that bind to the activated platelet surfaces are ready to receive direct binding from free emicizumab in the fluid.

The model presented in this study suggests that there are 2 mechanistic improvements that might enhance the ability of a bispecific antibody to promote factor X activation: enhanced turnover (increased $k^{cat}$) and slightly tighter binding to factor X. These predicted improvements can be seen in new bispecific antibodies being developed as replacements for FVIII. NXT007 is a bispecific Ab developed by engineering emicizumab. Mutations to heavy and light chains were introduced and the resulting molecules screened for activity. The screening identified a higher activity molecule that had the same affinity for FIXa but bound FX 30- to 40-fold more tightly [4]. Interestingly, this increase in binding affinity is consistent with the value that our model predicts for the maximal effect on FXa generation. Mim8 (denecimig) is a bispecific antibody developed by independent screening for binding to FIXa and FX [27]. The FIXa-binding arm of Mim8 was subsequently engineered to promote increased FIXa catalytic activity, which resulted in an Ab with increased FX activation relative to emicizumab. This increased activation is consistent with our suggestion that increased catalytic activity increases reaction velocity; however, Mim8 has different antigen-binding regions, likely resulting in different values for multiple binding parameters in our model [5,27]. Thus, the activity of Mim8 cannot be directly mapped onto our existing model.

## Supplementary Material

MMC1

The online version contains supplementary material available at https://doi.org/10.1016/j.jtha.2025.07.002.
