# Supplementary material for: Mathematical analysis of emicizumab: affinity-driven complex formation and lipid-surface reactions
Source: J Thromb Haemost. Author manuscript; Available in PMC 2025 Sep 29. (PMC12477644; doi:10.1016/j.jtha.2025.07.002)
Supplement: MMC1 [file NIHMS2104735-supplement-MMC1.pdf]

# Supplemental Material for: Mathematical analysis of emicizumab: affinity-driven complex formation and lipid surface reactions, by Madrigal *et al.*

## 1 Supplemental Experimental Data

Factor Xa binding to lipids was assessed essentially as described in Mertens et al.[6] Buffer was 20 mM HEPES (pH 7.4), 150 mM NaCl, 0.2% polyethylene glycol, and 5 mM CaCl2. Briefly, 25 µL of lipid was added to 25 µL of either factor Xa or factor Xa preincubated with emicizumab. The 50 µL volume had 25 nM FXa, 50 µM emicizumab, and the indicated concentration of lipid. The reaction mixture was incubated for 5 minutes then centrifuged for 10 minutes at 20,000 xg in an Eppendorf 5417R centrifuge to pellet the lipid and lipid bound proteins. A 20 µL aliquot was removed from the supernatant and factor Xa not bound to lipid measured by the ability to cleave Pefachrome FXa. Data was expressed as the ratio of activity relative to the activity in the absence of lipid. Data from four independent experiments is shown below in Figure S1.

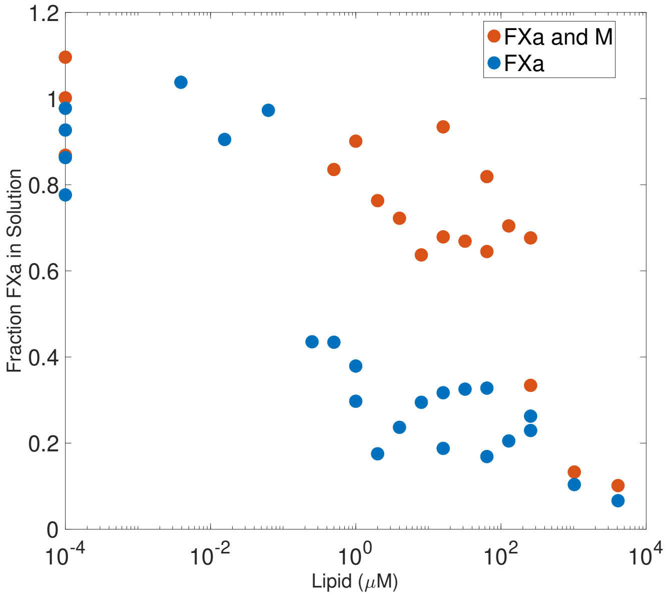


Figure S1: Experimental measurements of total fraction of solution-phase FXa concentration, i.e. free FXa and FXa in complex with emicizumab, in the absence of emicizumab (blue) and in the presence of emicizumab (orange) as a function of lipid concentration (0-4096$\mu$M).

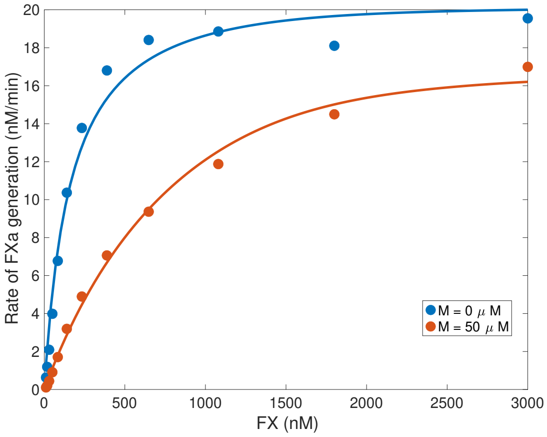


Figure S2: Experimental rate of FX activation by TF:VIIa (dots) as a function of FX (10-3000nM) concentration without emicizumab (blue) and with 50$\mu$M emicizumab (orange) with surface reaction velocity [4] fit (solid).

# 2 Supplemental Modeling Results: One-Arm Model

Here we detail the biochemical reaction scheme and kinetic rates for the One-Arm Model (Table S1). To address discrepancies in published values for the dissociation constant ($K_D$) of FX binding to emicizumab (X:M), we performed comparative modeling using two reported values: 55.8 nM from Mak et al. [5], and 1.85 $\mu$M from Kitazawa et al. [2, 1]. For each, we used a Markov-Chain Monte Carlo (MCMC) procedure to estimate the posterior distributions of the parameters $\alpha$ and $\beta$.

We observed a clear difference in model fit and posterior behavior between the two values of $K_D$, with much greater agreement between model and data using the Mak et al. value. (Compare Figure S3 with Figure S4). To quantitatively assess model performance, we evaluated the sum of squared relative residuals using the posterior mean estimates of $\alpha$ and $\beta$ from each fit. The model using the Mak et al. value of $K_D$ produced a substantially better fit, with a residual error of $18,617$ compared to $143,587$ for the Kitazawa value. Under the assumption of proportional error, this corresponds to a likelihood ratio test statistic of $\chi^2 \approx 204.3$ and a p-value smaller than $10^{-45}$, providing overwhelming statistical support for the Mak et al. value. We therefore proceed with this value in the modeling framework presented below.

Using this parameterization and the posterior means of $\alpha$ and $\beta$, we conducted a final set of simulations to evaluate different mechanistic hypotheses regarding X:M inhibition. (See Figure S5).

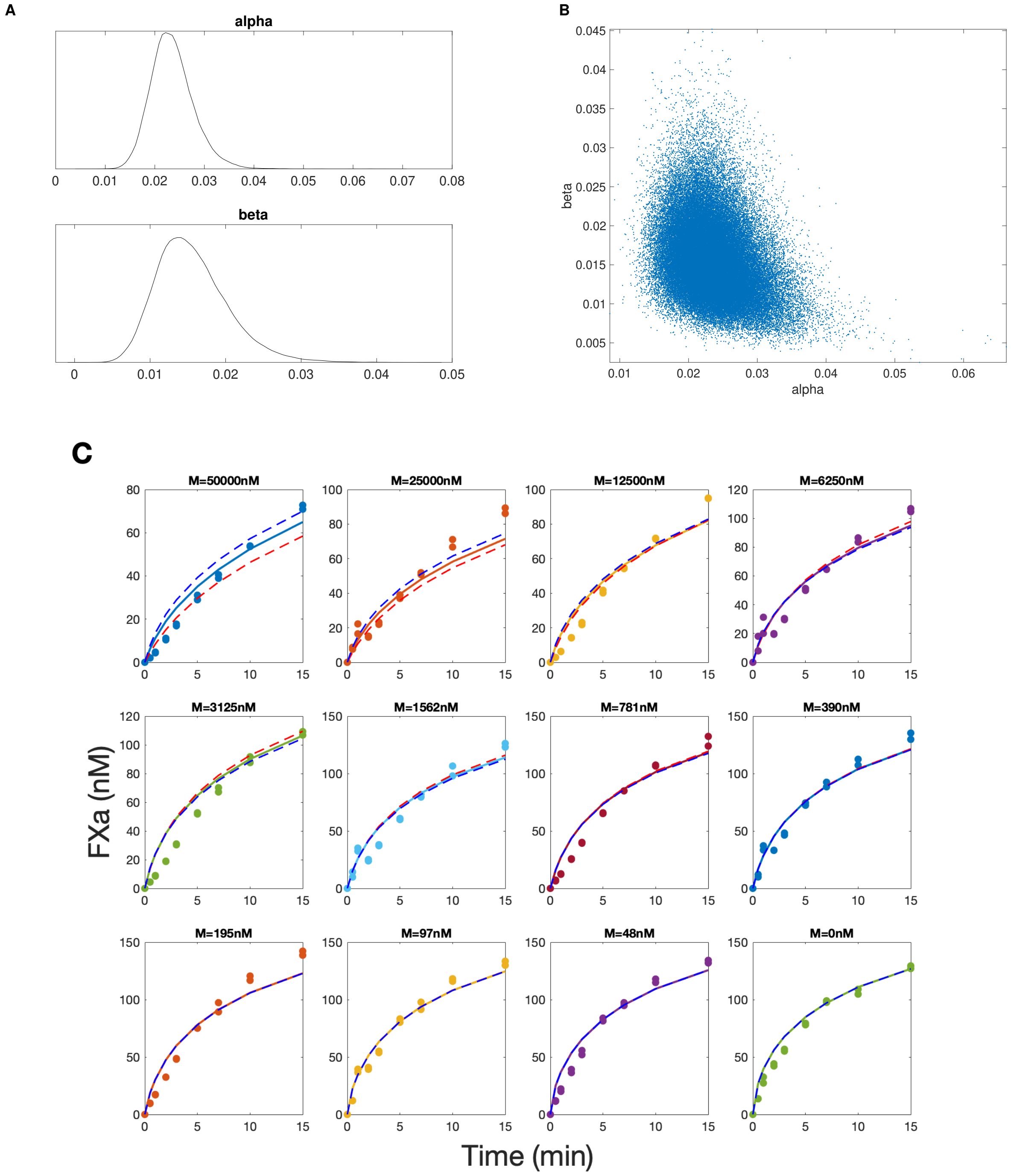


Figure S3: Results from the one-arm model MCMC simulations to estimate parameters $\alpha$ and $\beta$, using $K_D$ for X:M of 55 nM [5]. Parameters $\alpha$ and $\beta$ represent the scale factor on the association rate for X:M complex binding to lipid and to TF:VIIa, respectively. A) The posterior distribution of estimated parameters $\alpha$ and $\beta$ estimated with a 1-dimensional kernel density estimate. B) Scatter plot of pairwise relationship between estimates for $\alpha$ and $\beta$. C) Experimental FXa concentration (dots) generated through FX (175nM) activation by TF:VIIa (0.5nM) with lipid (80$\mu$M). One-arm model solution with mean estimated parameters $\alpha$ and $\beta$ (solid line).

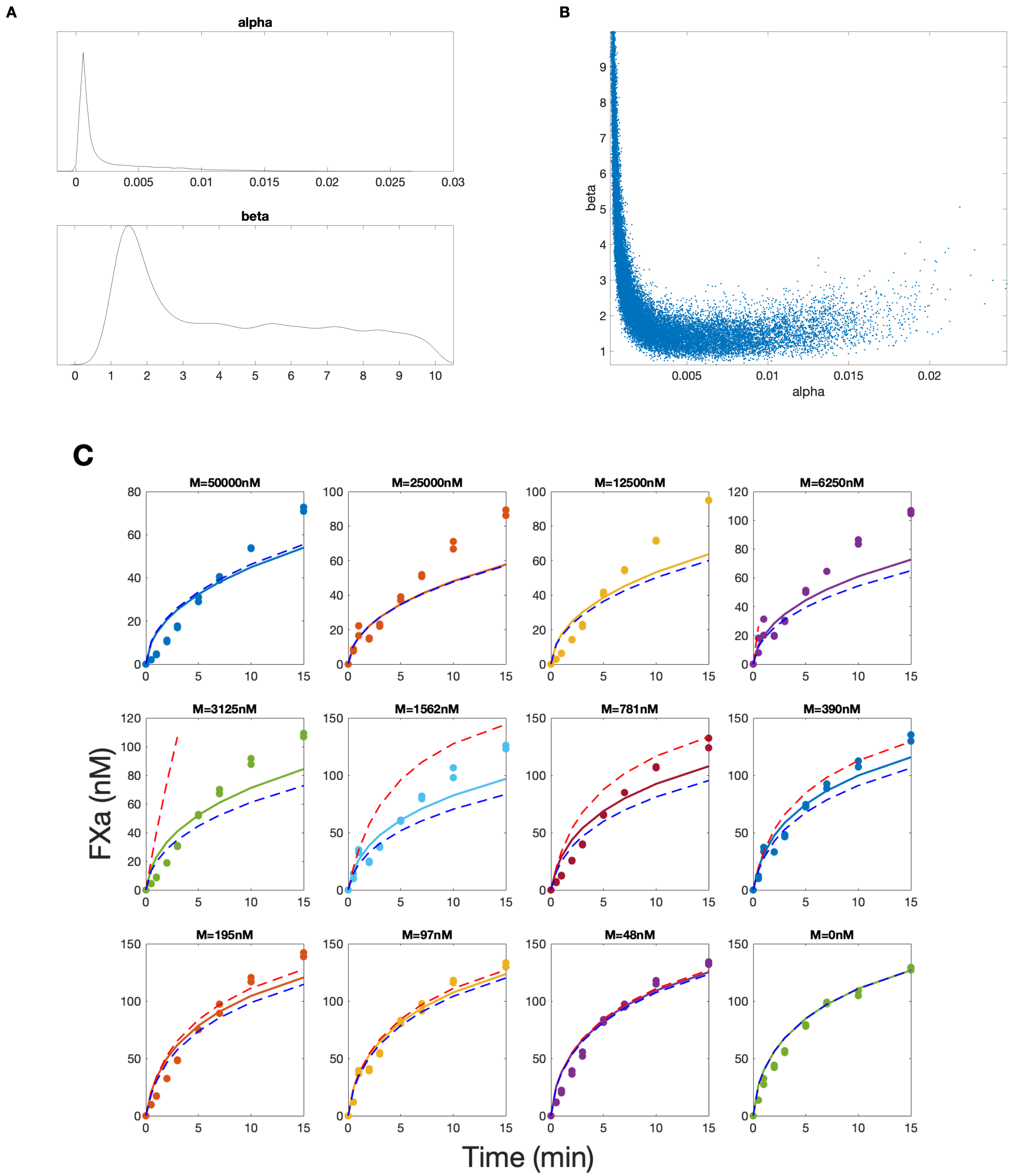


Figure S4: Results from the one-arm model MCMC simulations to estimate parameters $\alpha$ and $\beta$, using $K_D$ for X:M of 1.85 $\mu$M [2]. Parameters $\alpha$ and $\beta$ represent the scale factor on the association rate for X:M complex binding to lipid and to TF:VIIa, respectively. A) The posterior distribution of estimated parameters $\alpha$ and $\beta$ estimated with a 1-dimensional kernel density estimate. B) Scatter plot of pairwise relationship between estimates for $\alpha$ and $\beta$. C) Experimental FXa concentration (dots) generated through FX (175nM) activation by TF:VIIa (0.5nM) with lipid (80$\mu$M). One-arm model solution with mean estimated parameters $\alpha$ and $\beta$ (solid line).

| # | Reactants | Products | Parameters | $(nM^{-1}s^{-1})$ | $(s^{-1})$ | Note |
|---|---|---|---|---|---|---|
| 1 | $M{+}X^{b,+}$ | $M{:}X^{b,+}$ | $k^{+}_{MX^b}, k^{-}_{MX^b}$ | $4.63 \times 10^{-4}$ | $2.58 \times 10^{-2}$ | a |
| 2 | $M{+}X^{b,-}$ | $M{:}X^{b,-}$ | $k^{+}_{MX^b}, k^{-}_{MX^b}$ | $4.63 \times 10^{-4}$ | $2.58 \times 10^{-2}$ | a |
| 3 | $M{+}Xa^{b,+}$ | $M{:}Xa^{b,+}$ | $k^{+}_{MXa^b}, k^{-}_{MXa^b}$ | $4.63 \times 10^{-4}$ | $2.58 \times 10^{-2}$ | a |
| 4 | $M{+}Xa^{b,-}$ | $M{:}Xa^{b,-}$ | $k^{+}_{MXa^b}, k^{-}_{MXa^b}$ | $4.63 \times 10^{-4}$ | $2.58 \times 10^{-2}$ | a |
| 5 | $M{+}X$ | $M{:}X$ | $k^{+}_{MX}, k^{-}_{MX}$ | $4.63 \times 10^{-4}$ | $2.58 \times 10^{-2}$ | a |
| 6 | $M{:}X{+}L^{+}$ | $M{:}X^{b,+}$ | $\alpha \cdot k^{on}_{MX}, k^{off}_{MX}$ | $\alpha \cdot 0.01$ | 1.9 | b, e |
| 7 | $M{:}X{+}L^{-}$ | $M{:}X^{b,-}$ | $\alpha \cdot k^{on}_{MX}, k^{off}_{MX}$ | $\alpha \cdot 0.01$ | 1.9 | b, e |
| 8 | $M{+}Xa$ | $M{:}Xa$ | $k^{+}_{MXa}, k^{-}_{MXa}$ | $4.63 \times 10^{-4}$ | $2.58 \times 10^{-2}$ | a |
| 9 | $M{:}Xa{+}L^{+}$ | $M{:}Xa^{b,+}$ | $\alpha \cdot k^{on}_{MXa}, k^{off}_{MXa}$ | $\alpha \cdot 0.77$ | 3.3 | c, e |
| 10 | $M{:}Xa{+}L^{-}$ | $M{:}Xa^{b,-}$ | $\alpha \cdot k^{on}_{MXa}, k^{off}_{MXa}$ | $\alpha \cdot 0.77$ | 3.3 | c, e |
| 11a | $M{:}X^{b,+}{+}E^{b,+}$ | $M{:}X^{b,+}{:}E^{b,+}$ | $\beta \cdot k^{+}_{MX,E}, k^{-}_{MX,E}$ | $\beta \cdot 4.85$ | 15.56 | d |
| 11b | $M{:}X^{b,+}{:}E^{b,+}$ | $M{:}Xa^{b,+}{:}E^{b,+}$ | $k^{cat}_{M}$ | - | 22.56 | d |
| 12 | $M{:}Xa^{b,+}{+}E^{b,+}$ | $M{:}Xa^{b,+}{:}E^{b,+}$ | $k^{+}_{MXa,E}, k^{-}_{MXa,E}$ | 92.41 | 7.57 | d |
| 13 | $M{+}X^{b,+}{:}E^{b,+}$ | $M{:}X^{b,+}{:}E^{b,+}$ | $k^{+}_{M,XE}, k^{-}_{M,XE}$ | $4.63 \times 10^{-4}$ | $2.58 \times 10^{-2}$ | a |
| 14 | $M{+}Xa^{b,+}{:}E^{b,+}$ | $M{:}Xa^{b,+}{:}E^{b,+}$ | $k^{+}_{M,XaE}, k^{-}_{M,XaE}$ | $4.63 \times 10^{-4}$ | $2.58 \times 10^{-2}$ | a |

Table S1: One arm interaction model. List of reactions added to the two compartment lipid model with product inhibition. $M$ represents emicizumab. $E$ represents TF:VIIa. The superscripts $b,+$ and $b,-$ indicated bound to TF:VIIa$^{+}$ lipid and TF:VIIa$^{-}$ lipid, respectively. Kinetic rates taken from a) Binary binding of emicizumab to FX[5], $K_D = 55.8nM$. b) Binding of FX to lipid[8], $K_D = 190nM$. c) Binding of FXa to lipid[3, 4], $K_D = 4.2nM$ d) Activation of FX by TF:VIIa[4]. e) See Table S2 for $\alpha$ and $\beta$ values.

| Estimated Parameter | Mean ± std | note |
|---|---|---|
| $\alpha$ | $2.35 \times 10^{-2} \pm 4.30 \times 10^{-3}$ | a |
| $\beta$ | $1.56 \times 10^{-2} \pm 4.74 \times 10^{-3}$ | b |

Table S2: Estimated kinetic parameters of the one-arm model with $K_D$ for X:M of 55 nM [5] (see Figure S3. a) $\alpha$ scales the kinetic rates for FX/Xa-emicizumab complex lipid binding: $k^{on}_{MX} = \alpha k^{on}_{MX}$, $k^{on}_{MXa} = \alpha k^{on}_{MXa}$ b) $\beta$ scales the kinetic rates for FX/Xa-emicizumab complex binding TF:VIIa: $k^{+}_{MX,E} = \beta k^{+}_{MX,E}$, $k^{+}_{MXa,E} = \beta k^{+}_{MXa,E}$

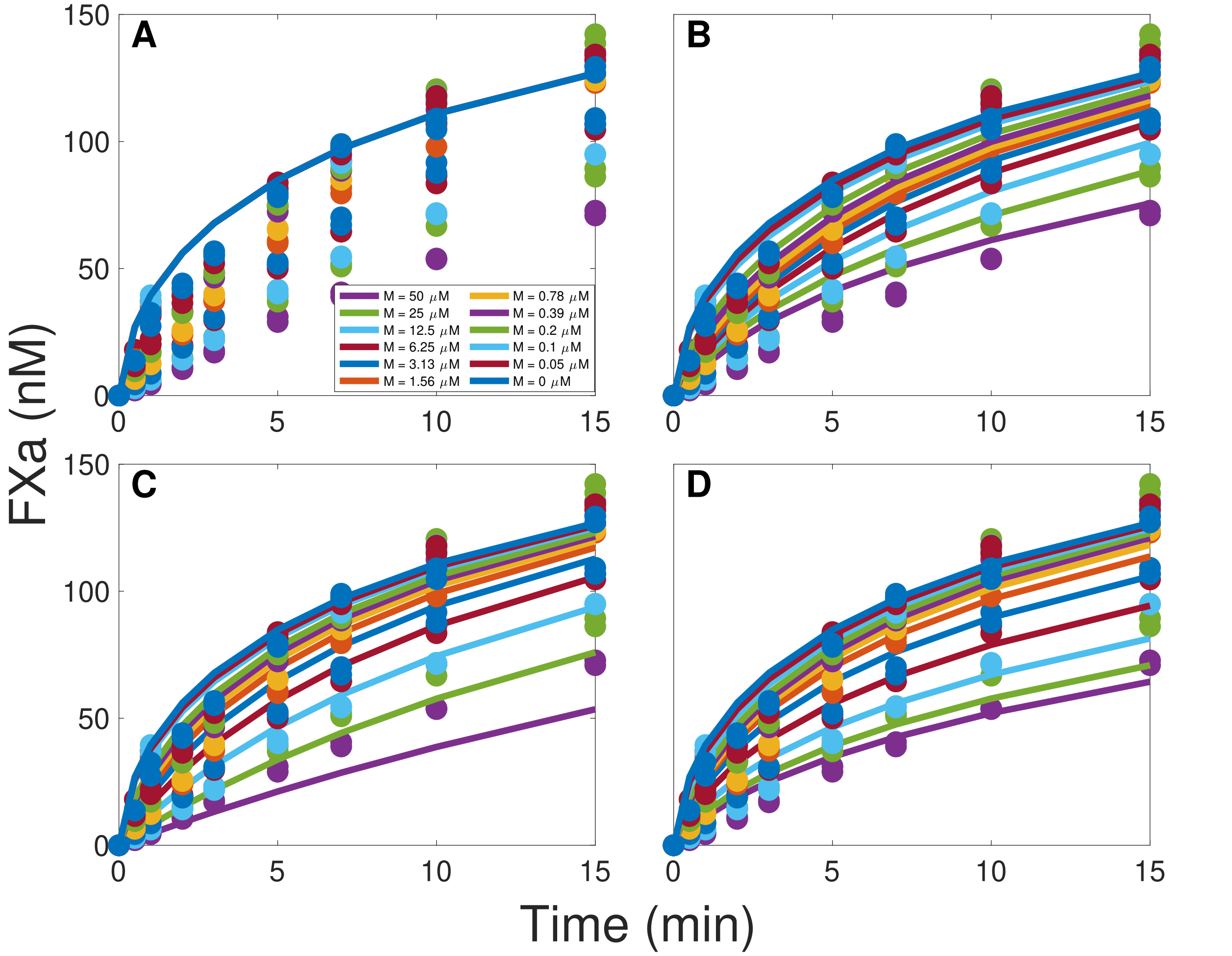


Figure S5: Final comparison of one-arm model with experimental results, assuming various mechanisms of inhibition. Experimental FX activation for varying emicizumab (M) concentrations are shown as dots and all model simulations are in solid lines. A) Using rates assumed from literature, and allowing maximal binding of X:M to lipid and TF:VIIa. B: Model simulations assuming that X:M is inhibited from binding to lipid. C: Model simulationsassuming that X:M is inhibited from binding TF:VIIa. D: Model assuming that X:M can be inhibited from both binding lipid and TF:VIIa.

# 3 Supplemental Modeling Results: Two-Arm Model

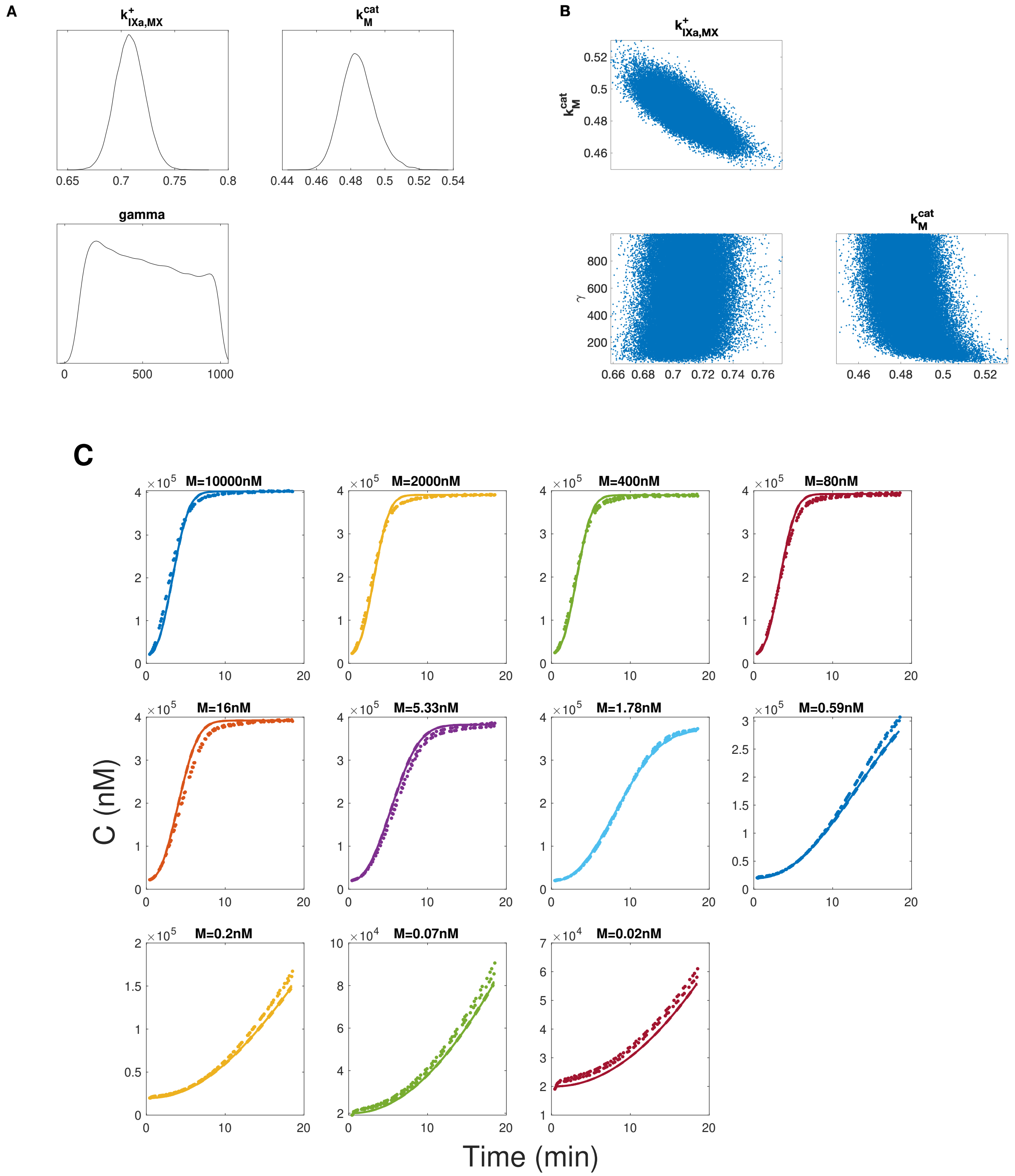


Figure S6: Results from the two-arm model MCMC simulations. A) The posterior distribution of estimated parameters $k^+_{IXa,MX}$, $k^{cat}_M$, and $\gamma$ estimated with a 1-dimensional kernel density estimate. B) Scatter plot of pairwise relationship between estimates for $k^+_{IXa,MX}$, $k^{cat}_M$, and $\gamma$. C) Experimental concentration of chromophore C (dots) generated through FX (140nM) activation by FIXa (1nM) with lipid (80$\mu$M). Two-arm model solution with mean estimated parameters $k^+_{IXa,MX}$, $k^{cat}_M$, and $\gamma$ (solid line).

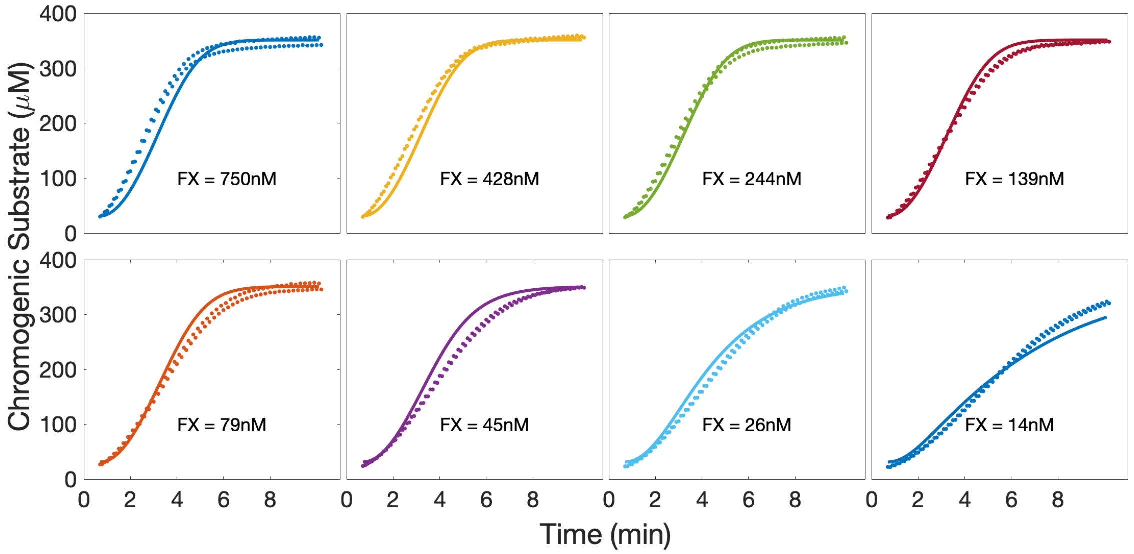


Figure S7: FX activation by FIXa in the presence of lipid and emicizumab: 400 $\mu$M emicizumab, varied FX, and 1 nM IXa. Each plot displays experimental measurements of cleavage of FXa chromogenic substrate (dots, duplicate data) for different concentrations of FX, overlaid with simulations of cleavage of FXa substrate generated with the two-arm mathematical model (solid lines).

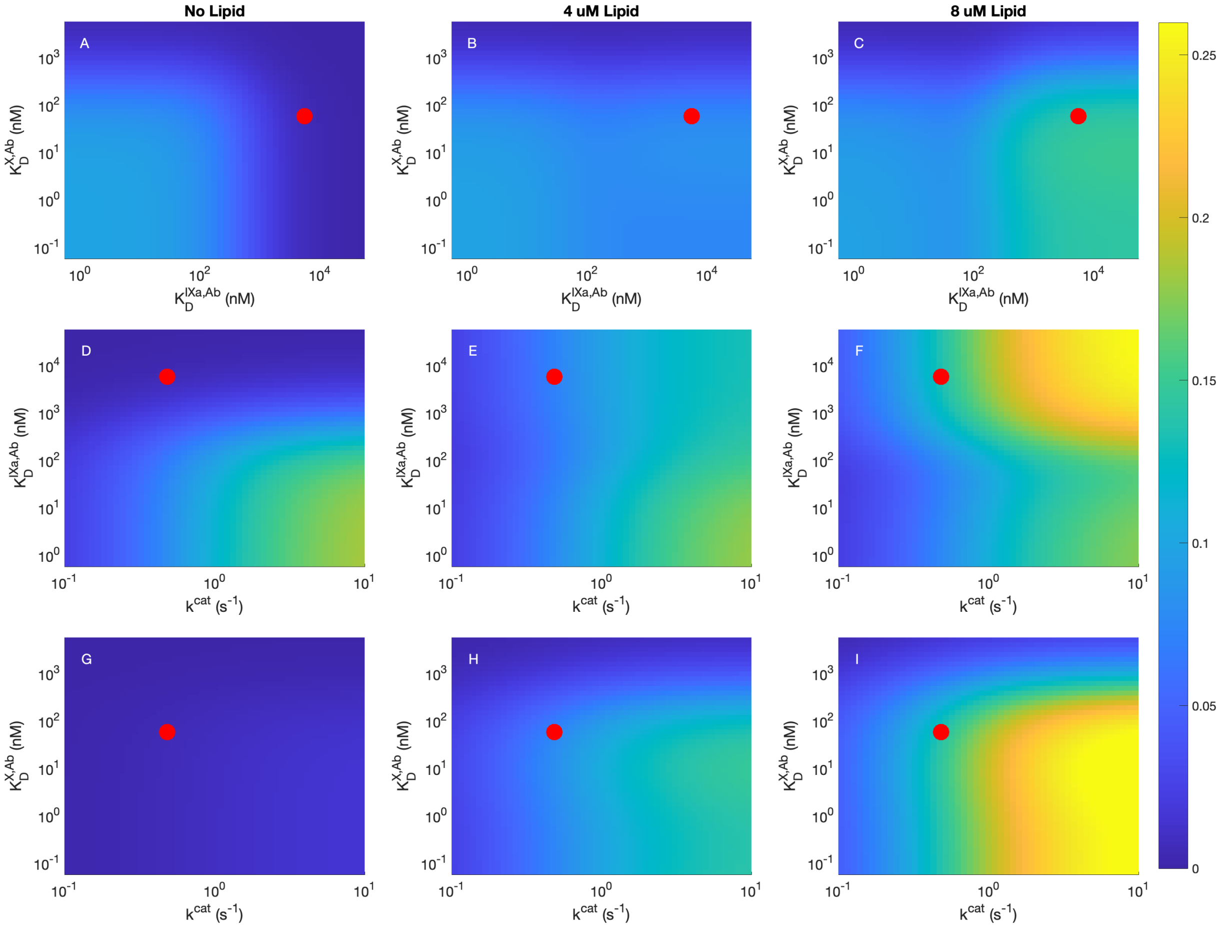


Figure S8: Heatmaps of the simulated reaction velocity, computed after 2 minutes of simulated reaction time, as a function of $K_D^{IXa,Ab}$, $K_D^{M,Ab}$, $k^{cat}$, and lipid concentration. Heatmaps of the simulated reaction velocity as $K_D^{IXa,Ab}$ and $K_D^{M,Ab}$ are varied with no lipid (A), 4 $\mu$M lipid (B), and 8 $\mu$M lipid (C). Heatmaps of the simulated reaction velocity as $K_D^{IXa,Ab}$ and $k^{cat}$ are varied with no lipid (D), 4 $\mu$M lipid (E), and 8 $\mu$M lipid (F). Heatmaps of the simulated reaction velocity as $K_D^{X,Ab}$ and $k^{cat}$ are varied with no lipid (G), 4 $\mu$M lipid (H), and 8 $\mu$M lipid (I). In every row, increasing lipid increases the velocity for some part of the parameter space. Plots in C,F, and I are the same as in the main manuscript and are reprinted here for comparison with the lower concentrations of lipid. Collectively, these plots show that, relative to the kinetic rate constants for emicizumab, increasing the $k^{cat}$ increases the velocity – in some cases almost doubling it – while decreasing $K_D^{M,Ab}$ and increasing $K_D^{IXa,Ab}$ give very minor increases in velocity.

| # | Reactants | Products | Parameters | $(nM^{-1}s^{-1})$ | $(s^{-1})$ | note |
|---|---|---|---|---|---|---|
| 1 | $IXa+L$ | $IXa^b$ | $k^{on}_{IXa}, k^{off}_{IXa}$ | 0.01 | 1.9 | a |
| 2 | $X+L$ | $X^b$ | $k^{on}_{X}, k^{off}_{X}$ | 0.01 | 1.9 | a |
| 3 | $Xa+L$ | $Xa^b$ | $k^{on}_{Xa}, k^{off}_{Xa}$ | 0.7 | 3.3 | b |
| 4a | $IXa^b+X^b$ | $IXa^b:X^b$ | $k^{+}_{X:IXa}, k^{-}_{X:IXa}$ | 0.01 | estimated | c |
| 4b | $IXa^b:X^b$ | $IXa^b+Xa^b$ | $k^{cat}$ | - | estimated | - |
| 5 | $IXa+M$ | $IXa:M$ | $k^{+}_{IXa:M}, k^{-}_{IXa:M}$ | $1.27\times10^{-4}$ | $70.2\times10^{-2}$ | d |
| 6 | $IXa^b+M$ | $IXa^b:M$ | $k^{+}_{IXa:M^b}, k^{-}_{IXa:M^b}$ | $1.27\times10^{-4}$ | $70.2\times10^{-2}$ | d |
| 7 | $X+M$ | $M:X$ | $k^{+}_{M:X}, k^{-}_{M:X}$ | $4.63\times10^{-4}$ | $2.58\times10^{-2}$ | d |
| 8 | $X^b+M$ | $M:X^b$ | $k^{+}_{M:X^b}, k^{-}_{M:X^b}$ | $4.63\times10^{-4}$ | $2.58\times10^{-2}$ | d |
| 9 | $Xa+M$ | $M:Xa$ | $k^{+}_{M:Xa}, \gamma\cdot k^{-}_{M:Xa}$ | $4.63\times10^{-4}$ | $\gamma\cdot2.58\times10^{-2}$ | d |
| 10 | $Xa^b+M$ | $M:Xa^b$ | $k^{+}_{M:Xa^b}, k^{-}_{M:Xa^b}$ | $4.63\times10^{-4}$ | estimated | e |
| 11 | $IXa:M+L$ | $IXa^b:M$ | $\alpha\cdot k^{on}_{IXa:M^b}, k^{off}_{IXa:M^b}$ | 0.01 | 1.9 | a, g |
| 12 | $M:X+L$ | $M:X^b$ | $\alpha\cdot k^{on}_{M:X^b}, k^{off}_{M:X^b}$ | 0.01 | 1.9 | a, g |
| 13 | $M:Xa+L$ | $M:Xa^b$ | $\alpha\cdot k^{on}_{M:Xa^b}, k^{off}_{M:Xa^b}$ | 0.7 | 3.3 | b, g |
| 14 | $IXa^b:X^b+M$ | $IXa^b:M:X^b$ | $k^{+}_{CM}, k^{-}_{CM}$ | $7.52\times10^{-4}$ | $3.73\times10^{-2}$ | d |
| 15 | $IXa^b+M:X^b$ | $IXa^b:M:X^b$ | $k^{+}_{IXa,M:X}, k^{-}_{IXa,M:X}$ | estimated | $47.9\times10^{-2}$ | d |
| 16 | $IXa^b:M+X^b$ | $IXa^b:M:X^b$ | $k^{+}_{IXa:M,X}, k^{-}_{IXa:M,X}$ | $5.16\times10^{-4}$ | $2.93\times10^{-2}$ | d |
| 17 | $IXa^b:M:X^b$ | $IXa^b:M:Xa^b$ | $k^{cat}_{M}$ | - | estimated | - |
| 18a | $IXa^b:M:Xa^b$ | $IXa^b:M+Xa^b$ | $k^{-}_{IXa:M,Xa}$ | - | estimated | e |
| 18b | $IXa^b:M:Xa^b$ | $IXa^b+M:Xa^b$ | $k^{-}_{IXa,M:Xa}$ | - | $47.9\times10^{-2}$ | d |
| 19a | $Xa+S:C$ | $Xa:S:C$ | $k_1, k_2$ | 0.01 | 1025.3 | f |
| 19b | $Xa:S:C$ | $Xa:S+C$ | $k_3$ | - | 78.7 | f |
| 20 | $Xa+S$ | $Xa:S$ | $k_1, \alpha_{CS}\cdot k_2$ | 0.01 | 2563 | f |
| 21a | $Xa^b+S:C$ | $Xa^b:S:C$ | $k_1, k_2$ | 0.01 | 1025.3 | f |
| 21b | $Xa^b:S:C$ | $Xa^b:S+C$ | $k_3$ | - | 78.7 | f |
| 22 | $Xa^b+S$ | $Xa^b:S$ | $k_1, \alpha_{CS}\cdot k_2$ | 0.01 | 2563 | f |
| 23a | $M:Xa+S:C$ | $M:Xa:S:C$ | $k_1, k_2$ | 0.01 | 1025.3 | f |
| 23b | $M:Xa:S:C$ | $M:Xa:S+C$ | $k_3$ | - | 78.7 | f |
| 24 | $M:Xa+S$ | $M:Xa:S$ | $k_1, \alpha_{CS}\cdot k_2$ | 0.01 | 2563 | f |
| 25 | $M+Xa:S$ | $M:Xa:S$ | $k^{+}_{M:Xa}, \gamma\cdot k^{-}_{M:Xa}$ | $4.63\times10^{-4}$ | $\gamma\cdot2.58\times10^{-2}$ | d,e |
| 26a | $M:Xa^b+S:C$ | $M:Xa^b:S:C$ | $k_1, k_2$ | 0.01 | 1025.3 | f |
| 26b | $M:Xa^b:S:C$ | $M:Xa^b:S+C$ | $k_3$ | - | 78.7 | f |
| 27 | $M:Xa^b+S$ | $M:Xa^b:S$ | $k_1, \alpha_{CS}\cdot k_2$ | 0.01 | 2563 | f |
| 28 | $M+Xa^b:S$ | $M:Xa^b:S$ | $k^{+}_{M:Xa}, \gamma\cdot k^{-}_{M:Xa}$ | $4.63\times10^{-4}$ | $\gamma\cdot2.58\times10^{-2}$ | d,e |
| 29 | $IXa:M+X$ | $IXa:M:X$ | $k^{+}_{M:X}, k^{-}_{M:Xa}$ | $4.63\times10^{-4}$ | $2.58\times10^{-2}$ | d |
| 30 | $IXa+M:X$ | $IXa:M:X$ | $k^{+}_{IXa:M}, k^{-}_{IXa:M}$ | $1.27\times10^{-4}$ | $70.2\times10^{-2}$ | d |
| 31 | $IXa:M:X$ | $IXa:M:Xa$ | $k^{cat}_{M}$ | - | estimated | - |

Table S3: Two arm interaction model. List of reactions and parameters. Gray rows represent reactions in the solution phase, white (no color) rows are reactions on lipid. $M$ represents emicizumab. The superscript $b$ indicates bound to lipid. Kinetic rates taken from a) Binding of FX to lipid[8], $K_D = 190nM$. b) Binding of FXa to lipid[3, 4], $K_D = 4.2nM$ c) $k^{+}_{IXa:X}$ assumed d) Binary binding of emicizumab to FX[5], $K_D = 55.8nM$; Binary binding of emicizumab to FIXa[5], $K_D = 5.53\mu M$; Ternary binding of emicizumab-FX to FIXa[5];Binary binding of FIXa-emicizumab to FX[5], $K_D = 56.7nM$; Ternary binding of emicizumab to FIXa-FX, $K_D = 49.6$; e) Assume equal to the kinetic rate for emicizumab unbinding FXa, $k^{-}_{M:Xa}$. f) Interactions with chromogenic substrate[7] where $\alpha_{CS}$ is the constant of proportionality. g) See Table S3 for $\alpha$, $\beta$ and $\gamma$ values.

| Estimated Parameter | Mean $\pm$ std | note |
|---|---|---|
| $K_D(nM)$ | $774.85 \pm 12.5$ | a |
| $k^{cat}(s^{-1})$ | $5 \times 10^{-3} \pm 7.7 \times 10^{-5}$ | - |
| $\gamma$ | $517.26 \pm 263.4$ | b |
| $k^{+}_{IXa,M:X}(nM^{-1}s^{-1})$ | $0.7082 \pm 0.0138$ | c |
| $k^{cat}_{M}(s^{-1})$ | $0.4845 \pm 0.01$ | - |

Table S4: Estimated kinetic parameters of the two arm interaction model. a) $k^{+}_{IXa:X} = 0.01$ assumed b) $\gamma$ scales the kinetic rate for binary unbinding of emicizumab from FXa, $k^{-}_{M:Xa} = \gamma k^{-}_{M:Xa}$, $K_D = 28824$nM; c)Ternary binding of emicizumab-FX to FIXa[5], $K_D = 0.68$nM

# References

[1] Kitazawa, T., Esaki, K., Tachibana, T., Ishii, S., Soeda, T., Muto, A., Kawabe, Y., Igawa, T., Tsunoda, H., Nogami, K., Shima, M., and Hattori, K. (2017). Factor VIIIa-mimetic cofactor activity of a bispecific antibody to factors IX/IXa and X/Xa, emicizumab, depends on its ability to bridge the antigens. *Thrombosis and Haemostasis*, 117(7):1348–1357.

[2] Kitazawa, T., Igawa, T., Sampei, Z., Muto, A., Kojima, T., Soeda, T., Yoshihashi, K., Okuyama-Nishida, Y., Saito, H., Tsunoda, H., Suzuki, T., Adachi, H., Miyazaki, T., Ishii, S., Kamata-Sakurai, M., Iida, T., Harada, A., Esaki, K., Funaki, M., Moriyama, C., Tanaka, E., Kikuchi, Y., Wakabayashi, T., Wada, M., Goto, M., Toyoda, T., Ueyama, A., Suzuki, S., Haraya, K., Tachibana, T., Kawabe, Y., Shima, M., Yoshioka, A., and Hattori, K. (2012). A bispecific antibody to factors IXa and X restores factor VIII hemostatic activity in a hemophilia A model. *Nature Medicine*, 18(10):1570–1574. Publisher: Nature Publishing Group.

[3] Krishnaswamy, S. (1992). The interaction of human factor VIIa with tissue factor. *Journal of Biological Chemistry*, 267(33):23696–23706.

[4] Madrigal, J., Monroe, D. M., Sindi, S. S., and Leiderman, K. (2024). Modeling the distribution of enzymes on lipid vesicles: A novel framework for surface-mediated reactions in coagulation. *Mathematical Biosciences*, 374:109229.

[5] Mak, S., Marszal, A., Matscheko, N., and Rant, U. (2023). Kinetic analysis of ternary and binary binding modes of the bispecific antibody emicizumab. *mAbs*, 15(1):2149053. Publisher: Taylor & Francis _eprint: https://doi.org/10.1080/19420862.2022.2149053.

[6] Mertens, K., Cupers, R., Van Wijngaarden, A., and Bertina, R. M. (1984). Binding of human blood-coagulation Factors IXa and X to phospholipid membranes. *Biochemical Journal*, 223(3):599–605.

[7] Stobb, M. T., Monroe, D. M., Leiderman, K., and Sindi, S. S. (2019). Assessing the impact of product inhibition in a chromogenic assay. *Analytical Biochemistry*, 580:62–71.

[8] Van De Waart, P., Bruls, H., Hemker, H. C., and Lindhout, T. (1983). Interaction of bovine blood clotting factor Va and its subunits with phospholipid vesicles. *Biochemistry*, 22(10):2427–2432.
